# The Effect of Chinese Herbal Medicine Formula mKG on Allergic Asthma by Regulating Lung and Plasma Metabolic Alternations

**DOI:** 10.3390/ijms18030602

**Published:** 2017-03-10

**Authors:** Meng Yu, Hong-Mei Jia, Feng-Xia Cui, Yong Yang, Yang Zhao, Mao-Hua Yang, Zhong-Mei Zou

**Affiliations:** Institute of Medicinal Plant Development, Chinese Academy of Medical Sciences and Peking Union Medical College, Beijing 100193, China; yumeng.5555@163.com (M.Y.); 18911566112@126.com (H.-M.J.); fengxiacui@163.com (F.-X.C.); dsz200200@163.com (Y.Y.); youngzhao91@163.com (Y.Z.); mhyangmh@foxmail.com (M.-H.Y.)

**Keywords:** allergic asthma, mKG, *Sophora flavescens*, *Glycyrrhiza uralensis*, Angelica oil, metabolomics, biomarkers, UPLC-Q-TOF/MS

## Abstract

Asthma is a chronic inflammatory disorder of the airway and is characterized by airway remodeling, hyperresponsiveness, and shortness of breath. Modified Kushen Gancao Formula (mKG), derived from traditional Chinese herbal medicines (TCM), has been demonstrated to have good therapeutic effects on experimental allergic asthma. However, its anti-asthma mechanism remains currently unknown. In the present work, metabolomics studies of biochemical changes in the lung tissue and plasma of ovalbumin (OVA)-induced allergic asthma mice with mKG treatment were performed using ultra high-performance liquid chromatography coupled with quadrupole time-of-flight mass spectrometry (UPLC-Q-TOF/MS). Partial least squares–discriminate analysis (PLS−DA) indicated that the metabolic perturbation induced by OVA was reduced after mKG treatment. A total of twenty-four metabolites involved in seven metabolic pathways were identified as potential biomarkers in the development of allergic asthma. Among them, myristic acid (**L3** or **P2**), sphinganine (**L6** or **P4**), and lysoPC(15:0) (**L12** or **P16**) were detected both in lung tissue and plasma. Additionally, l-acetylcarnitine (**L1**), thromboxane B2 (**L2**), 10-HDoHE (**L10**), and 5-HETE (**L11**) were first reported to be potential biomarkers associated with allergic asthma. The treatment of mKG mediated all of those potential biomarkers except lysoPC(15:0) (**P16**). The anti-asthma mechanism of mKG can be achieved through the comprehensive regulation of multiple perturbed biomarkers and metabolic pathways.

## 1. Introduction

Asthma is a chronic inflammatory disorder of the airway characterized by airway remodeling, hyperresponsiveness, and shortness of breath [1,2]. Currently, three types of prescription medications, including inhaled short and long-acting β2 agonists, inhaled and oral corticosteroids, and leukotriene antagonists are usually adopted for the treatment of asthma patients [3]. However, these treatments are not effective for all patients and also demonstrate significant, reproducible, undesirable side effects of systemic corticosteroids in the therapeutic response [4]. Accordingly, the search for new anti-asthma drugs is an area of intense research activity. More recently, Chinese medicines and their formulas have aroused much interest especially due to their low side effects in the treatment of asthma [5].

Modified Kushen Gancao Formula (mKG), derived from traditional Chinese herbal medicines (TCM), has been demonstrated to show prominent anti-inflammatory effects and good therapeutic effects on pulmonary fibrosis in our previous study [6]. It contains three herbs: viz., the roots of *Sophora flavescens* Ait. (Ku-Shen), the rhizome of *Glycyrrhiza uralensis* Fisch. (Gan-Cao), and Angelica oil. Ku-Shen is a commonly used TCM, used mainly in combination with other medicinal plants in prescriptions to treat fever, dysentery, jaundice, eczema, asthma, and inflammatory disorders [7]. Recent studies have revealed that the extract of Ku-Shen suppresses both helper T cell 2 (Th2) and tumor necrosis factor-α (TNF-α) associated inflammation in murine asthma models [8]. The primary effective components of Ku-Shen are matrine and oxymatrine, and both have been demonstrated to show significant anti-asthmatic effects in OVA-induced allergic asthma mouse models [9,10]. Gan-Cao is a very common herbal medicine used in almost half of Chinese herbal formulas to reduce toxicity, enhance effectiveness, and improve the flavor of other herbs [11]. It has been reported that Gan-Cao extract has obvious anti-asthma effects, which is associated with the inhibition of the production of IgE in OVA-sensitized asthma mice [12]. The primary constituents of Gan-Cao are glycyrrhizin, and a mixture of calcium and potassium salts of glycyrrhizinic acid which represent the efficacy on asthmatic features via attenuating inflammation effects in asthma mouse models [13,14]. In addition, Ku-Shen and Gan-Cao are also the major herbs in ASHMI™ (Anti-asthma the Herbal Medicine Intervention), which can prevent allergic asthma airway hyper-reactivity in mice and inhibits acetylcholine (ACh) induced airway smooth muscle (ASM) contraction in tracheal rings from allergic asthmatic mice [15]. Angelica oil is considered to be the main biologically active component in the roots of *Angelica sinensis* (Oliv.) Diels and exhibits many bioactivities, especially anti-inflammatory, immunostimulatory, and anti-cancer effects [16]. Preclinical studies have indicated that Angelica oil suppresses airway smooth muscle contraction and is often used to treat asthma and other allergic diseases [17]. The main chemical constituents of Angelica oil include Z-ligustilide, butylphthalide, and senkyunolide A. Combination therapies of TCM prescription have been validated and show potential clinical benefits. To achieve a better therapeutic effect, the three Chinese herbs are combined in a single prescription mKG to treat allergic asthma. More research is needed to investigate the effectiveness and the mechanism of the herbal formula.

Metabolomics, the comprehensive analysis of small biochemicals in biological systems, can provide insight into complex biochemical processes and enable the identification of biomarkers that may serve as therapeutic targets [18]. Successful applications of the metabolomic approach have been located at active herb molecules, single herb preparations, and multiple herb derived prescriptions [19,20]. Therefore, utilizing a metabolomics platform to explore disease-relevant metabolic profile changes and discover potential biomarkers can provide valuable information for a deeper understanding of the pathological mechanisms of disease and drug treatment, especially in complex diseases.

The OVA-induced allergic asthma mouse model is becoming increasingly popular as an animal model of allergic asthma to elucidate asthma pathology and evaluate new therapeutic agents. Correspondingly, our recent metabolomics investigation of the plasma has further revealed promising allergic asthma-related metabolic biomarkers from the mouse model of OVA-induced allergic asthma [21]. Metabolites in lung tissue could directly reflect the pathophysiological state, and potentially generate unique perspectives for understanding disease mechanisms. Therefore, in this paper the metabolic profiles of the lung tissue and plasma in the mouse model of OVA-induced allergic asthma with and without treatment by mKG were investigated using UPLC-Q-TOF/MS to provide a comprehensive understanding of the underlying mechanism of mKG against allergic asthma.

## 2. Results

### 2.1. Identification of the Chemical Constituents in mKG Extract

**Figure 1:**
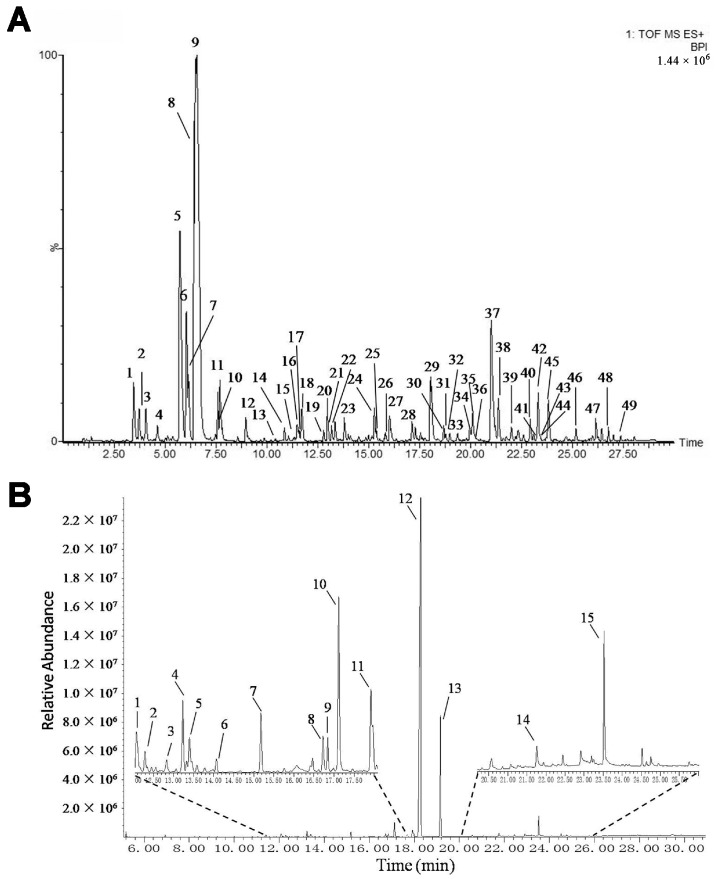
BPI chromatogram of mKG extract (**A**) in positive ion mode analyzed by UPLC-Q-TOF/MS, and TIC chromatogram of Angelica oil (**B**) analyzed by using GC-MS.

The mKG is composed of Kushen Gancao extract and Angelica oil. The Kushen Gancao extract samples were analyzed using the UPLC-Q-TOF/MS method. The base peak intensity (BPI) chromatograms of mKG, Kushen, and Gancao extracts in positive ion electrospray ionization (ESI) mode are shown in Figure 1A and Figure S1. The MS data showed high precision with all the mass accuracies within 5 ppm, which provided valuable information for confirming the accurate molecular weight and composition of the constituents. Forty-nine compounds were tentatively identified on the basis of their retention behaviors, accurate molecular weight, and MS^E^ fragment data, or by comparison with reference substances or literature data (chemical structures are shown in Figure S2, corresponding quasi-molecular ions and their fragment ions in the MS^E^ spectra are listed in Table S1).

The Angelica oil was analyzed by the gas chromatography-mass spectrometer (GC-MS) method. The total ion chromatograms (TIC) of Angelica oil is shown in Figure 1B. A total of 15 compounds were identified based on reference substances or literature data (Figure S3 and Table S2).

### 2.2. Histopathology

**Figure 2:**
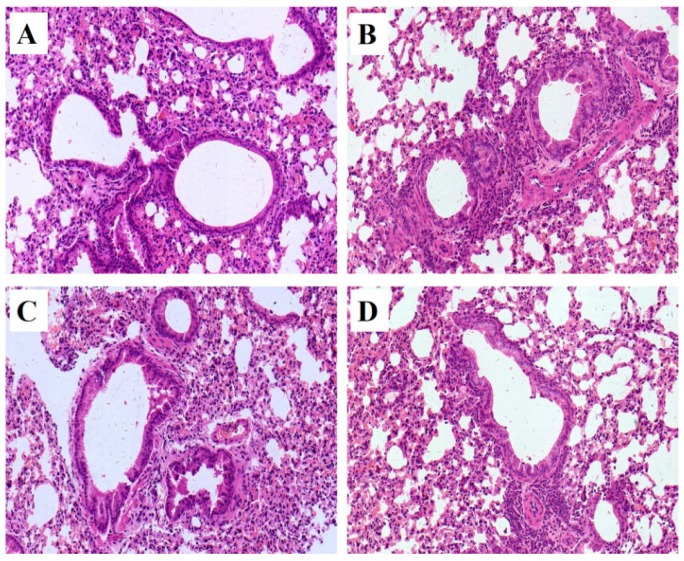
Example of pathological changes of the lung tissue observed by Hematoxylin and Eosin (HE) staining (Light microscopy, ×400): (**A**) (control); (**B**) (model); (**C**) (Dexamethasone (2 mg·kg^−1^)); (**D**) (mKG (9.7 g·kg^−1^)).

Pathological changes of lung tissue were assessed by hematoxylin-eosin (HE) staining of the paraffin embedded section (Figure 2). All pathological abnormalities in OVA-induced allergic asthma mice, such as infiltration of inflammatory cells into perivascular and connective tissues, lumen narrowing, and mucosa thickening, were markedly ameliorated by both mKG and dexamethasone pretreatments. The histopathological results demonstrated that mKG and dexamethasone showed a similar anti-asthma effect on the pathological changes with OVA-challenged allergic asthma mice (Figure 2C,D).

### 2.3. Lung Tissue Metabolic Profiles of OVA-Induced Allergic Asthma with mKG Treatment

**Figure 3:**
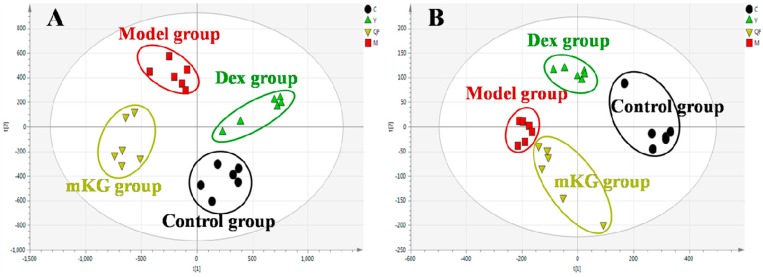
Score plots from the PLS−DA model classifying the control group (●), model group (■), mKG group (▼), and Dex group (▲) with mice lung tissue samples in positive (**A**) and negative (**B**) ion modes by UPLC-Q-TOF/MS.

Metabolic profiles of the lung tissue in each group were analyzed by UPLC-Q-TOF/MS in positive and negative modes. The typical BPI chromatograms of all experimental groups are shown in Figure S4. A supervised PLS−DA model was performed for the analysis of the normalization spectral data from all the experimental groups. Meanwhile, we used seven-fold cross-validation to validate the statistical significance for the PLS−DA model. The parameters for the classification of the lung metabolic profiles both in positive and negative ion modes are shown in Table 1, which support good prediction analysis. From the PLS−DA score plot (Figure 3A,B), it was demonstrated that the metabolic profile of the OVA-induced mice was far from that of normal mice, suggesting that significant biochemical changes were induced by OVA. Meanwhile, a clear separation among the control, model, mKG, and dexamethasone treated groups were observed. The treatments of mKG and dexamethasone could regulate the metabolic deviations induced by the experimental asthma. However, their regulations were different. The mKG group was closer to the control group than the dexamethasone group in the first principal component in the positive ion mode, while closer to the control group in the second principal component in the negative ion mode.

### 2.4. Identification of Potential Lung Tissue Biomarkers Associated with OVA-Induced Asthma

**Figure 4:**
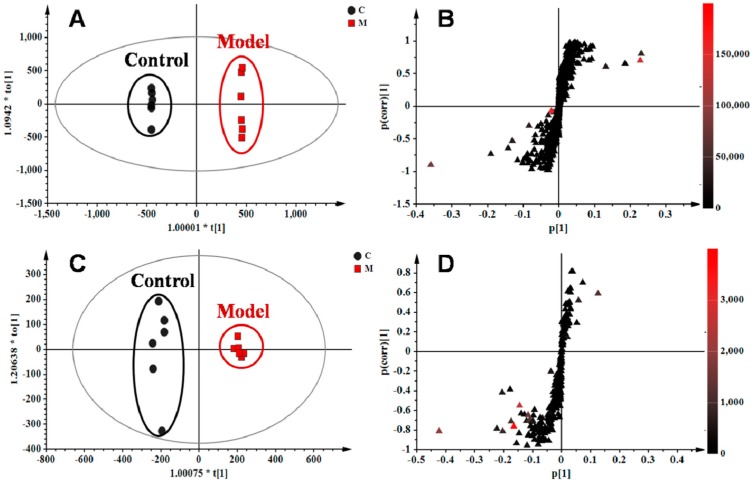
OPLS−DA score plots and *S*-plots based on lung tissue of the control and model groups in (**A**,**B**) *R*^2^*X* = 0.554, *R*^2^*Y* = 1.000, *Q*^2^ (cum) = 0.907) positive and (**C**,**D**) *R*^2^*X* = 0.558, *R*^2^*Y* = 0.990, *Q*^2^ (cum) = 0.953) negative ion modes.

The orthogonal partial least squares–discriminate analysis (OPLS−DA) method was employed to sharpen an already established separation between the model and control groups in PLS−DA. The *S*-plot and variable importance of project (VIP) were used to select potential biomarkers (Figure 4). The *S*-plots based on lung metabolic profiles between the control group and model group indicated that 13 ions contributed to the clustering, with retention time and *m*/*z* pairs of 0.63_204.1233, 4.15_393.2245, 4.79_246.2432, 5.19_355.2626, 5.20_373.2735, 6.41_302.3056, 7.12_522.3552, and 8.95_256.2637 in positive ion mode and 5.19_407.2790, 6.93_343.2262, 7.04_319.2264, 7.04_480.3081, and 8.91_327.2315 in negative ion mode. Their VIP values are listed in Table 2. Among those ions, some of them had close retention times and signified the same metabolites such as ions 5.19_355.2626 and 5.20_373.2735. Identification of the metabolites was performed based on the accurate mass and the collected MS^E^ spectra measurements via Q-TOF/MS and comparison of the data was performed with authentic reference materials and database resources. Databases, such as Human Metabolome Database (HMDB), METLIN, MassBank, and Kyoto Encyclopedia of Genes and Genomes (KEGG) were used for confirmation. As a result, 11 metabolites in the lung tissue samples were identified as potential biomarkers related to OVA-induced asthma. They are: l-acetylcarnitine (**L1**), thromboxane B2 (**L2**), myristic acid (**L3**), cholic acid fragments (**L4**,**L5**), dihydrosphinganine (**L6**), LysoPC(18:1(11Z)) (**L7**), palmitic amide (**L8**), cholic acid (**L9**), 10-HDoHE (**L10**), 5-HETE (**L11**), LysoPC(15:0) (**L12**), and docosahexaenoic acid (DHA) (**L13**) (Table 2). Among them, 10 metabolites were identified using MS spectral databases and one metabolite (**L9**) was confirmed using the reference substance (Figure S5). Moreover, four of them (**L1**, **L2**, **L10,** and **L11**) were first reported to have potential relevance to the pathogenesis of allergic asthma. mKG mediated the deviations of all biomarkers, while dexamethasone did not show any effects on LysoPC(15:0) (**L12**).

### 2.5. Plasma Metabolic Profiles of OVA-Induced Allergic Asthma with mKG Treatment

**Figure 5:**
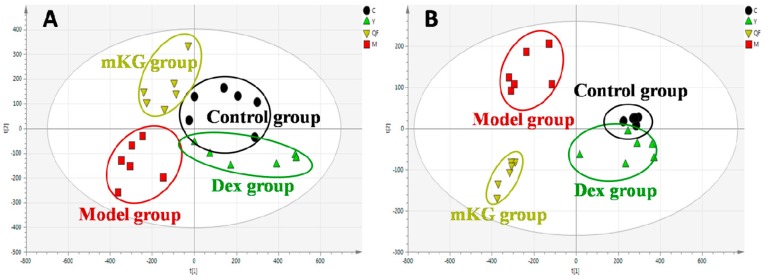
Score plots from the PLS−DA model classifying the control group (●), model group (■), mKG group (▼), and Dex group (▲) with mice plasma samples in positive (**A**) and negative (**B**) ion modes by UPLC-Q-TOF/MS.

The metabolic profiles of the plasma samples from OVA-induced allergic asthma mice with and without mKG treatment were characterized using UPLC-Q-TOF/MS in both positive and negative ion scan modes. PLS−DA was used to evaluate the metabolic patterns of OVA-induced mice with mKG treatment (Figure 5A,B). The parameters of the PLS−DA models for the classification and the change trends of potential biomarkers in the plasma samples are shown in Table 1 and Table 3. Among these plasma metabolites, 12 metabolites were identified by the MS spectral databases and four metabolites (**P12**–**15**) were confirmed using the reference substances (Figures S6–S9). The metabolic profile of mice in the mKG treated group differed from the model group and were closer to the control group, indicating that the deviations induced by OVA were significantly improved after mKG treatment. The results showed that mKG was much closer to the control group than that of the dexamethasone treated group in positive ion mode.

### 2.6. Perturbed Metabolic Pathways in Response to Allergic Asthma and mKG Treatment

**Figure 6:**
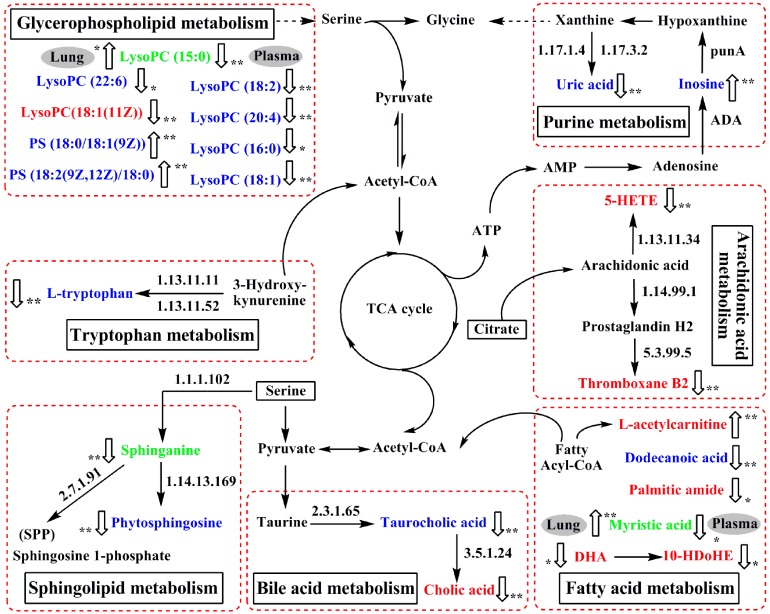
The perturbed network of the potential biomarker associated allergic asthma and the modulation of mKG according to the KEGG database. 1.17.1.4: xanthine dehydrogenase; 1.17.3.2: xanthineoxidase; ADA: Adenosine deaminase; 1.13.11.11: tryptophan 2,3-dioxygenase (TDO); 1.13.11.52: indoleamine 2,3-dioxygenase (IDO); 1.13.11.34: arachidonate 5-lipoxygenase; 1.14.99.1: prostaglandin-endoperoxide synthase; 1.1.1.102: dehydrosphinganine reductase (DSR); 5.3.99.5: thromboxane-A synthase; 2.7.1.91: sphingosine kinase; 1.14.13.169: sphingolipid C4-monooxygenase (SUR2); 2.3.1.65: bile acid-CoA:amino acid N-acyltransferase (BAT); 3.5.1.24: choloylglycine hydrolase. Metabolites measured in lung tissue were labeled in red, metabolites labeled in blue were measured in plasma, and metabolite names in green mean they were measured both in plasma and lung tissue. 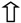: up-regulated, 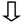: down-regulated; * *p* < 0.05, ** *p* < 0.01.

Based on the identified potential biomarkers of OVA-induced allergic asthma from the lung tissue and plasma of mice, the metabolic network affected by the OVA-induced mice was depicted according to the KEGG (http://www.genome.ad.jp/kegg/) and the HMDB (http://www.hmdb.ca/) (Figure 6). The results indicated that seven metabolic pathways were disturbed in OVA-induced allergic asthma mice, including fatty acid metabolism (**P1**, **P2**, **L1**, **L3**, **L8**, **L10** and **L13**), sphingolipid metabolism (**P3**, **P4**, and **L6**), glycerophospholipid metabolism (**P5**–**P11**, **P16**, **L7**, and **L12**), purine metabolism (**P12** and **P13**), tryptophan metabolism (**P14**), bile acid metabolism (**P15**, **L4**, **L5**, and **L9**), and arachidonic acid metabolism (**L2** and **L11**).

#### 2.6.1. Fatty Acid Metabolism

It is known that fatty acids are important constituents of cell membranes and the largest energy reserves in the body derive from fatty acid oxidation. During the fatty acid oxidation, the l-carnitine cycle is an essential step for mitochondrial fatty acid oxidation, in which the fatty acyl CoA enters the mitochondria as fatty acylcarnitines via carnitine transport [22]. As a result, acylcarnitines are generated during this carnitine-dependent pathway and play an important role through the mitochondrial membrane. It has been reported that the inhibition of fatty acid oxidation results in the accumulation of acylcarnitine derivatives [23]. In our study, the levels of l-acetylcarnitine (**L1**) was increased in the lung tissue of model mice compared with the control group, most likely because the fatty acid oxidation is suppressed, which led to the metabolic remodeling to meet the energy requirements in the body. Palmitic amide (**L8**), a fatty acid amide, competes with endocannabinoids to bond with the active site of the enzyme fatty acid amide hydrolase, which could increase the concentration of endocannabinoids in the fatty acid metabolism [24]. An increased release of the endogenous cannabinoid in response to inhalation of an allergen in asthmatic patients has been reported [25]. Although high levels of endocannabinoids were not detected in our study, decreased levels of palmitic amide (**L8**) were observed in the lung tissue of model mice. Whether palmitic amide (**L8**) combined with the fatty acid amide hydrolase enzyme and degraded in the formation of allergic asthma needs to be further investigated. Docosahexaenoic acid (DHA) (**L13**) and the metabolite of 10-HDoHE (**L10**) are commonly thought to be anti-inflammatory nutrients, with protective effects in inflammatory diseases including asthma and allergies [26]. The lower level of docosahexaenoic acid (DHA) (**L13**) and 10-HDoHE (**L10**) in the lung tissue of OVA-induced model mice indicated the inflammatory reactions in the formation of allergic asthma. The role of dodecanoic acid (**P1**) in fatty acid metabolism is to mediate cell signaling and energy storage. A recent report has shown that dodecanoic acid (**P1**) exhibits anti-inflammatory activities through the inhibition of NF-κB activation and the phosphorylation of the mitogen activated protein (MAP) kinases [27]. In this study, decreased dodecanoic acid (**P1**) in the plasma of model mice was observed, suggesting the body’s excessive consumption of dodecanoic acid (**P1**) for suppressing the NF-κB activation and the phosphorylation of MAP kinases, and thus it participates in the body’s response to inflammation. Additionally, a previous study reports that asthmatic patients consume higher myristic acid in their daily energy intake [28]. Of particular interest, in our current study, myristic acid (**P2** or **L3**) has been detected both in the plasma and lung tissue, which decreased in plasma but increased in lung tissue. Although this difference has not yet been elucidated, the result suggests significant associations of myristic acid (**P2** or **L3**) with allergic asthma. Both treatments of mKG and dexamethasone mediated the deviation of **P1**, **P2** (**L3**), **L1**, **L8**, **L10**, and **L13** induced by OVA, suggesting that mKG has a similar regulation effect on fatty acid metabolism to that of dexamethasone.

#### 2.6.2. Sphingolipid Metabolism

Sphingolipids play significant physiological roles in cell proliferation, cell-cell interaction, differentiation, and apoptosis [29]. Phytosphingosine (**P3**), metabolized from sphinganine (**P4** or **L6**) by 3-dehydrosphinganine reductase (DSR), prevents the opening of the mitochondrial permeability transition pore (MPT) in the sphingolipid metabolism [30]. Meanwhile, sphinganine (**P4** or **L6**) could generate the intracellular sphingolipid metabolite of sphingosine 1-phosphate (SPP) by sphingosine kinase in response to numerous stimuli. SPP acts as an extracellular lipid modulator in human airway smooth muscle cell functions that promotes inflammation and airway remodeling in asthma [31]. The decreased phytosphingosine (**P3**) and sphinganine (**P4**) in the model group might accelerate the accumulations of SPP, which could then lead to inflammation cell proliferation in the development of allergic asthma. The treatments with mKG and dexamethasone significantly reversed the decreasing levels of phytosphingosine (**P3**) and sphinganine (**P4**), suggesting both mKG and dexamethasone could effectively ameliorate the abnormal change of sphingolipid metabolism.

#### 2.6.3. Glycerophospholipid Metabolism

Glycerophospholipids are important components of the cell membrane structure. The disturbance of glycerophospholipid metabolism would result in the alterations of energy metabolism, inflammation, as well as endothelial dysfunction, and would be related to the disruptions of cell membranes and the occurrence of inflammation [32,33]. In this study, the concentrations of nine glycerophospholipids were found to be changed in the OVA-induced allergic asthma model group. The levels of PS (18:0/18:1(9Z)) (**P10**), PS (18:2(9Z,12Z)/18:0) (**P11**), and lysoPC(15:0) (**L12**) were up-regulated, whereas lysoPC(22:6) (**P5**), lysoPC(18:2) (**P6**), lysoPC(20:4) (**P7**), lysoPC(16:0) (**P8**), lysoPC(18:1) (**P9**), lysoPC(15:0) (**P16**), and lysoPC(18:1(11Z)) (**L7**) were down-regulated compared with the normal group. Among them, lysoPC(15:0) (**P16** or **L12**) was detected both in the plasma and lung tissue, with a low level of it in the former and a high level in the latter. These results suggested that perturbation of glycerophospholipid metabolism might be involved in the pathophysiology of allergic asthma. With the treatment of mKG, all derivations of metabolites except lysoPC(15:0) (**P16**) were corrected, indicating that the regulation of the perturbation of glycerophospholipid metabolism contributes to the anti-asthma effect of mKG. However, dexamethasone treatment did not regulate the lysoPC(20:4) (**P7**) and lysoPC(15:0) (**L12**). These findings revealed that more disturbed metabolites related to glycerophospholipid metabolism could be regulated comprehensively by mKG rather than by dexamethasone.

#### 2.6.4. Purine Metabolism

Purine metabolism plays an important role in energy metabolism, which generates a series of metabolites such as adenosine monophosphate (AMP), hyoxanthine, xanthine, uric acid, and inosine. Those molecules are proportional to neutrophilic inflammation of chronic and obstructive pulmonary disease [34,35]. In our previous study, we confirmed that uric acid significantly decreased and inosine increased in relation to OVA-induced allergic asthma mice [21]. Here, mKG and dexamethasone reversed the decreased level of uric acid (**P12**) and the increased level of inosine (**P13**), suggesting that the interventions of mKG and dexamethasone play a key role against purine metabolism disorder in the development of allergic asthma.

#### 2.6.5. Tryptophan Metabolism

l-tryptophan (**P14**) is an essential amino acid that serves as a critical precursor in the energy metabolism of an organism. It has been reported that systemic tryptophan and its catabolites relate to the severity of rhinovirus-induced asthma exacerbation in patients with allergic asthma [36]. In our study, a low level of l-tryptophan (**P14**) was observed in the plasma of OVA-challenged mice, and this depletion of l-tryptophan (**P14**) led to abnormalities of the energy metabolism. After the treatments of mKG and dexamethasone, the decreased level of l-tryptophan (P14) could be up-regulated, indicating that mKG has a similar regulation effect on tryptophan metabolism as compared to dexamethasone.

#### 2.6.6. Bile Acid Metabolism

Cholic acid (**L9**), the metabolite of taurocholic acid (**P15**) by choloylglycine hydrolase, is significantly reduced and related to inflammatory cells and cytokines in bronchoalveolar lavage fluid (BALF) with the house dust mite (HDM)-induced allergic asthma [37]. Although the relationship between taurocholic acid (**P15**) and asthma has not been elucidated yet, in this study, decreased concentrations of cholic acid (**L9**) in lung tissue and taurocholic acid (**P15**) in plasma were observed in the OVA-induced model group compared with the control group. This suggests that choloylglycine hydrolase may be inhibited in the formation of allergic asthma. mKG treatment up-regulated the levels of cholic acid (**L9**) and taurocholic acid (**P15**) in allergic asthma, which is in agreement with the observed fact that mKG significantly improved the dysfunction of bile acid metabolism induced by OVA.

#### 2.6.7. Arachidonic Acid Metabolism

Arachidonic acid (AA), an important polyunsaturated fatty acid (PUFA), is an indispensable precursor of various lipid mediators and participates in many physiological and pathophysiological processes. AA can be oxygenated by lipoxygenases (LOs) and further transformed into a complex mixture of oxygenated product hydroxyeicosatetraenoic acids (HETEs), which mediate or modulate inflammatory reactions in the airway hyperreactivity involved in asthma, especially 5-HETE [38]. Accordingly, the decreased levels of 5-HETE (**L11**) are associated with inflammatory reactions in the development of allergic asthma. Thromboxanes, generated from AA via the cyclooxygenase pathway, play an important role in platelet aggregation and thrombosis in patients with asthma [39]. The lower level of thromboxane B2 (**L2**) was observed in the lung tissue of model mice, which provided evidence to support the inhibition of the activities of the cyclooxygenase in the formation of OVA-induced allergic asthma. In the mKG treated group, 5-HETE (**L11**) and thromboxane B2 (**L2**) were up-regulated compared with the model group, similar to that of the dexamethasone treatment, suggested that mKG could effectively ameliorate the abnormal changes of arachidonic acid metabolism.

## 3. Discussion

Multiple genetic and environmental factors contribute to the phenotypic expression of asthma. For those patients with persistent symptoms of asthma, anti-inflammatory treatment with inhaled corticosteroids remains the first-line treatment. Yet some patients are nonresponsive and undesirable side effects of systemic corticosteroids occur in the therapeutic response to steroid treatment. Therefore, the search for more effective anti-asthma drugs with low side effects is desperately needed. mKG is composed of a famous Chinese herb that has been advocated for thousands of years by TCM physicians for dealing with disease, traditional medical Kushen Gancao Formula and Angelica oil. The combination therapy of TCM prescription was a unique medical system which can hit multiple targets with multiple components. It has an overall therapeutic effect on allergic asthma. In this study, the pharmacodynamics and mechanism of mKG were explored in an OVA-induced mouse allergic asthma model using UPLC-Q-TOF/MS based metabolomics.

Existing metabolomics that have studied allergic asthma have uncovered altered metabolism and metabolic profiles in serum, urine, and BALF samples from clinical and experimental asthma [40,41,42], but it has not directly investigated the relevant lung metabolome changes, which would provide novel insights into changes in lung metabolism under asthmatic conditions. Correspondingly, our recent study has used metabolomics to analyze the plasma from OVA-induced allergic asthma, and demonstrated that this approach could offer opportunities for disease-related biomarker and pathway discovery [21]. In this study, an integrated approach combining the lung tissue and blood metabolic analysis has the potential to be a useful and convenient method to investigate the therapeutic effect of mKG in a mouse model of OVA-induced allergic asthma.

Our present investigation successfully uncovered concurrent OVA-induced metabolic profiling changes in the lung tissue and plasma. We identified several consistent metabolic signatures, including 18 potential allergic asthma biomarkers relating to fatty acid metabolism, glycerophospholipid metabolism, purine metabolism, and bile acid metabolism, which strongly corroborated previous metabolomics studies [21,40,41,42]. It is worth mentioning that the lung tissue encompasses unique disease-relevant metabolic signatures in OVA-induced allergic asthma. Here, a prominent metabolic feature observed was an alteration to arachidonic acid metabolism in the lung tissue, which has not been previously reported in asthma. Arachidonic acid metabolism may follow multiple interrelated pathways, leading to the generation or release of a wide variety of inflammation substances, which mediate or modulate inflammatory reactions [38]. Decreases in 5-HETE (**L11**) and thromboxane B2 (**L2**) suggested that perturbation of arachidonic acid metabolism is involved in the pathophysiology of allergic asthma.

To understand the multi-targeted effect of mKG against allergic asthma in depth, metabolites in lung tissue and plasma were measured using UPLC-Q-TOF/MS, and multivariate statistical analysis was performed by PLS−DA. With the multivariate analysis of the UPLC/MS data, the deviations of the metabolic profiles were significantly improved with the treatments of mKG and dexamethasone, suggesting that their anti-asthma effects are most likely to ameliorate the metabolic disorders induced by OVA. The therapeutic effect of mKG on allergic asthma may be involved in regulating the dysfunctions of fatty acid metabolism, sphingolipid metabolism, glycerophospholipid metabolism, purine metabolism, tryptophan metabolism, bile acid metabolism, and arachidonic acid metabolism. These metabolism pathways were found to be associated with OVA-induced allergic asthma in terms of the biochemical changes of metabolites. After treatment with mKG, all derivations of the 24 metabolites except lysoPC(15:0) (**P16**) were corrected. Meanwhile, mKG ameliorated the infiltration of inflammatory cells into perivascular and connective tissues, lumen narrowing, and mucosa thickening in the histopathological study. These findings indicated that active components in the mKG group exist that regulate different disorder networks in the pathogenesis of allergic asthma.

Collectively, these results suggest that some asthma-associated metabolic alterations detected by UPLC-Q-TOF/MS are reflected both in the lung tissue and bloodstream. mKG not only ameliorated the pathological changes, but also synergistically mediated the abnormalities of the metabolic network, which may be helpful for understanding its mechanism of action and selecting candidate anti-asthma herbal formulas for future clinical evaluation.

## 4. Materials and Methods

### 4.1. Chemicals and Reagents

HPLC-grade acetonitrile was bought from Merck (Darmstadt, Germany). Formic acid (HPLC grade) was obtained from Tedia (Fairfield, CA, USA). Dexamethasone, ammonium formate, aluminium hydroxide, and leucine-enkephalin were purchased from Sigma Aldrich (St. Louis, MO, USA). Pure water (18.2 MΩ) was prepared with a Milli-Q water purification system (Millipore, Paris, France). All other used chemicals were of analytical grade.

### 4.2. Raw Herbal Medicines and mKG Extract

Ku-Shen, Gan-Cao, and Dang-Gui were obtained from Beijing Tongren Tang Pharmaceutical Co. Ltd. (Beijing, China). All these raw herbs were identified as the roots of *Sophora flavescens* Ait., the rhizome of *Glycyrrhiza uralensis* Fisch, and the roots of *Angelica sinensis* (Oliv.) Diels. by Professor Yulin Lin of the Institute of Medicinal Plant Development (IMPLAD), Chinese Academy of Medical Sciences and Peking Union Medical College. The specimens of the above three herbs are preserved in the National Compound Library of TCM in IMPLAD. The mKG extract was prepared in our laboratory [6].

### 4.3. Qualitative Characteristics of Chemical Constituents in mKG Extract

Identification of chemical constituents in the Kushen Gancao extract was performed by UPLC-Q-TOF/MS analysis and the Angelica oil was analyzed by using the GC-MS method. The UPLC-MS spectra of samples were acquired in positive mode. The optimized UPLC-MS and GC-MS conditions are shown in the Supplementary Materials.

### 4.4. Animal Treatment and Sample Collection

Twenty-four healthy, 4–6 weeks old, female BALB/c mice were obtained from the Institute of Laboratory Animal Science, CAMS & PUMC, Beijing, China. The mice were housed in cages and maintained (20–25 °C and 40%–60% relative humidity) under controlled conditions of 12 h light-12 h dark cycles with commercial diet and water available ad libitum. The animals were randomly divided into 4 groups: (1) control group; (2) model group; (3) positive control group treated with dexamethasone (Dex); (4) mKG treated group (mKG).

Mice (except control group) were sensitized and challenged with OVA to develop allergic airway inflammation. Briefly, mice were sensitized by intraperitoneal (i.p.) injection of 10 μg OVA, and then received 4 mg Al(OH)_3_ suspended in 0.2 mL saline on days 0, 7, and 14. From days 16–22, mice were challenged with 4% OVA aerosol for 30 min during the days 16–22, and the positive control group was orally gavaged dexamethasone at the dose of 2 mg·kg^−1^. The mKG treated group was administered mKG orally at a dose of 9.7 g·kg^−1^ once daily for 22 days. Saline aerosol was used as a negative control.

Mice were anesthetized 24 h after the last aerosol challenge and blood samples were collected with sodium heparin as an anticoagulant and centrifuged at 4000 rpm for 15 min at 4.0 °C, and the supernatants were stored at −80 °C until metabolomics analysis. The lung tissue were quickly removed, and the middle lobe of the right lung was cut and put into a flasket containing 10% buffered formalin solution for the pathological analysis, while the rest of the lung samples were frozen immediately in liquid nitrogen and stored at −80 °C for later analysis. All experimental procedures were approved by the Ethics Committee of the Institute of Medicinal Plant Development, CAMS & PUMC (SYXK 2013–0023).

### 4.5. Histopathology

To observe pathological changes of OVA-induced allergic airway inflammation in mice, the middle lobe of the right lung from each mouse was fixed in 10% buffered formalin solution for 48 h, embedded in paraffin, 5 μm sectioned, and stained with HE. Images were obtained and analyzed under light microscopy (Olympus Corp., Tokyo, Japan).

### 4.6. Sample Preparation for Metabolomics

Lung tissues were extracted using a two-step procedure. Each left lung tissue sample (30 mg) was homogenized in 2000 μL of the first chilled extraction solvent (a mixture of chloroform, methanol and water (1:2:1, *v*/*v*/*v*)) by a homogenizer (IKA, Staufen, Germany) at 30,000 rpm. Then the mixture was vortexed for 5 min, followed by centrifugation at 6000 rpm for 15 min at 4 °C. 1500 μL of the supernatant was aspirated into a fresh tube, and then the deposit was re-homogenized with ice cold methanol (2000 μL). 1500 μL aliquot of supernatant was transferred to the above tube after centrifugation for drying, and then the dried metabolites extract was dissolved in 300 μL of acetonitrile-water (1:1, *v*/*v*), and an aliquot of 2 μL was injected for UPLC/MS analysis.

### 4.7. Data Acquisition

The lung tissue samples were analyzed on a Waters Acquity™ Ultra Performance LC system (Waters Corporation, Milford, MA, USA) equipped with a BEH C18 column (100 mm × 2.1 mm, 1.7 μm). The autosampler temperature was kept at 4 °C and the column compartment was set at 40 °C. The mobile phase was composed of solvents A (2 mM ammonium acetate in 95% H_2_O/5% acetonitrile + 0.1% acetic acid) and B (2 mM ammonium acetate in 95% acetonitrile/5% H_2_O + 0.1% acetic acid). The linear gradient program for lung tissue samples: 0–0.5 min, 1% B; 0.5–2 min, 10% B; 2–5 min, 10%–60% B; 5–9 min, 60%–90% B; 9–12 min, 90%–100% B; 12–16 min, washing with 100% B, and 16–19 min, equilibration with 1% B with a rate of 0.45 mL/min.

A Waters SYNAPT G2 HDMS (Waters Corp., Manchester, UK) was used to carry out the mass spectrometry with an electrospray ionization source (ESI) operating in positive and negative ion modes, respectively. The parameters were set as previously described [43]. Capillary voltages, 3.0 KV (ES+) and 2.5 KV (ES−); sample cone voltage, 40 V; extraction cone voltage, 4.0 V. Used drying gas nitrogen, desolvation gas rate and temperature, 800 L/h and 400 °C; cone gas rate, 50 L/h; source temperature, 100 °C; scan time and inter scan delay, 0.15 and 0.02 s, respectively. Leucine-enkephalin was selected as the lockmass in all analyses (*m*/*z* 556.2771 for positive ion mode and *m*/*z* 554.2615 for negative ion mode) at a concentration 0.5 μg/mL with a flow rate of 5 μL/min. Centroid data was collected and the mass range was set from *m*/*z* 100 to *m*/*z* 1500.

### 4.8. Multivariate Analysis

The UPLC-Q-TOF/MS raw data were processed by MarkerLynx Applications Manager version 4.1 (Waters, Manchester, UK). The procedure included integration, normalization, and alignment of peak intensities, and a list of *m*/*z* and retention times with corresponding peaks was provided for all metabolites in every sample in the data set. The resulting data set was analyzed by PLS-DA using SIMCA-P software (version 13.0, Umetrics AB, Malmö, Sweden) after a Pareto-scaled procedure.

### 4.9. Statistical Analysis

One-way ANOVA was performed using the Statistical Package for Social Science program (SPSS 16.0, Chicago, IL, USA). *P* values less than 0.05 was considered statistically significant.

## 5. Conclusions

In the present work, the protective effect of mKG was evaluated in OVA-induced allergic asthma mice using a metabolomic approach. Twenty-four potential biomarkers were identified from lung tissue and plasma samples. Among them, three metabolites including myristic acid (**P2** or **L3**), sphinganine (**P4** or **L6**), and lysoPC(15:0) (**P16** or **L12**) were measured both in the lung tissue and plasma. However, their changes occurred in opposite directions. Low levels of the three metabolites in plasma and high levels in lung tissue were observed. Additionally, four metabolites were firstly reported as potential biomarkers of OVA-induced allergic asthma. mKG showed effects on all potential biomarkers except lysoPC(15:0) (**P16**). The anti-asthma mechanism of mKG may be involved in the regulation of the metabolic pathways including fatty acid metabolism, sphingolipid metabolism, glycerophospholipid metabolism, purine metabolism, tryptophan metabolism, bile acid metabolism, and arachidonic acid metabolism. This study suggests that mKG exerts synergistic therapeutic efficacies to allergic asthma and the experimental studies may be useful in selecting candidate anti-asthma herbal formulas for future clinical evaluation.

## Figures and Tables

**Table 1:** The parameters for assessing the modeling quality of PLS−DA in positive and negative ion modes of plasma and lung tissue samples.

Sample	Group	Components	PLS−DA (Positive Ion Mode)			PLS−DA (Negative Ion Mode)		
			*R*^2^*X*	*R*^2^*Y*	*Q*^2^ (cum)	*R*^2^*X*	*R*^2^*Y*	*Q*^2^ (cum)
Plasma samples	Control vs. Model	3	0.684	0.998	0.939	0.823	0.991	0.973
	Model vs. mKG	3	0.579	0.992	0.891	0.558	0.963	0.898
	Model vs. Dex	3	0.679	0.998	0.943	0.801	0.989	0.884
Lung tissue samples	Control vs. Model	3	0.417	0.992	0.894	0.558	0.990	0.919
	Model vs. mKG	3	0.613	0.991	0.848	0.753	0.985	0.939
	Model vs. Dex	3	0.464	0.987	0.836	0.587	0.970	0.977

**Table 2:** Potential biomarkers characterized in the lung tissue profile and their change trends in different groups.

NO.	Metabolites	RT (min)	*m*/*z*	Formula	VIP	M/C	Y/M	mKG/M	Pathways
Positive ion mode									
L1	l-Acetylcarnitine ^b^	0.63	204.1233	C_9_H_17_NO_4_	3.59	↑ **	↓ **	↓	Fatty acid metabolism
L2	Thromboxane B2 ^b^	4.15	393.2245	C_20_H_34_O_6_	3.39	↓ **	↑ *	↑	Arachidonicacid metabolism
L3	Myristic acid ^b^	4.79	246.2432	C_14_H_28_O_2_	13.30	↑ *	↓ **	↓	Fatty acid metabolism
L4	Cholic acid fragment ^a^	5.19	355.2626	C_24_H_34_O_2_	5.41	↓ **	↑ **	↑ **	Bile Acid Biosynthesis
L5	Cholic acid fragment ^a^	5.2	373.2735	C_24_H_36_O_3_	3.24	↓ **	↑	↑	Bile Acid Biosynthesis
L6	Sphinganine ^b^	6.41	302.3056	C_18_H_39_NO_2_	14.52	↑ **	↓ **	↓ *	Sphingolipid metabolism
L7	LysoPC(18:1(11Z)) ^b^	7.12	522.3552	C_26_H_52_NO_7_P	6.20	↓ **	↑	↑	Glycerophospholipid metabolism
L8	Palmitic amide ^b^	8.95	256.2637	C_16_H_33_NO	4.01	↓ *	↑ *	↑	Fatty acid metabolism
Negative ion mode									
L9	Cholic acid ^a^	5.19	407.279	C_24_H_40_O_5_	4.04	↓ **	↑ **	↑ **	Bile Acid Biosynthesis
L10	10-HDoHE ^b^	6.93	343.2262	C_22_H_32_O_3_	1.71	↓ *	↑ *	↑	Fatty acid metabolism
L11	5-HETE ^b^	7.04	319.2264	C_20_H_32_O_3_	7.90	↓ **	↑ **	↑ **	Arachidonicacid metabolism
L12	LysoPC(15:0) ^b^	7.04	480.3081	C_23_H_48_NO_7_P	2.33	↑ *	↑	↓	Glycerophospholipid metabolism
L13	Docosahexaenoic acid (DHA) ^b^	8.91	327.2315	C_21_H_31_O_2_	3.39	↓ *	↑	↑	Fatty acid metabolism

The M/C represents the change trend in the model group compared with the control group, Y/M and mKG/M represent the change trends in the positive group and mKG group compared with the model group, respectively. ^a^ Metabolites validated by reference standards; ^b^ Metabolites identified by databases. ↑: up-regulated, ↓: down-regulated; * *p* < 0.05, ** *p* < 0.01.

**Table 3:** Potential biomarkers characterized in the plasma profile and their change trends in different groups.

NO.	Metabolites	RT (min)	*m*/*z*	Formula	VIP	M/C	Y/M	mKG/M	Pathways
Positive ion mode									
P1	Dodecanoic acid ^b^	2.25	218.2116	C_12_H_24_O_2_	2.60	↓ **	↑ **	↑ **	Fatty acid metabolism
P2	Myristic acid ^b^	2.87	246.2427	C_14_H_28_O_2_	2.57	↓ **	↑ **	↑ **	Fatty acid metabolism
P3	Phytosphingosine ^b^	4.13	318.3002	C_18_H_39_NO_3_	5.00	↓ **	↑ **	↑ **	Sphingolipid metabolism
P4	Sphinganine ^b^	6.06	302.3053	C_18_H_39_NO_2_	4.70	↓ **	↑ **	↑ **	Sphingolipid metabolism
P5	LysoPC(22:6) ^b^	6.85	568.3402	C_30_H_50_NO_7_P	2.45	↓ **	↑ **	↑	Glycerophospholipid metabolism
P6	LysoPC(18:2) ^b^	6.90	520.3405	C_26_H_50_NO_7_P	8.34	↓ **	↑ **	↑ **	Glycerophospholipid metabolism
P7	LysoPC(20:4) ^b^	6.92	544.3402	C_28_H_50_NO_7_P	5.95	↓ **	↓	↑ **	Glycerophospholipid metabolism
P8	LysoPC(16:0) ^b^	7.85	496.3404	C_24_H_50_NO_7_P	5.56	↓ *	↑ *	↑	Glycerophospholipid metabolism
P9	LysoPC(18:1) ^b^	8.46	522.3554	C_26_H_52_NO_7_P	4.01	↓ **	↑ **	↑ *	Glycerophospholipid metabolism
P10	PS(18:0/18:1(9Z)) ^b^	13.57	790.5587	C_42_H_80_NO_10_P	1.79	↑ **	↓ *	↓ *	Glycerophospholipid metabolism
P11	PS(18:2(9Z,12Z)/18:0) ^b^	13.67	788.5428	C_42_H_78_NO_10_P	3.64	↑ **	↓ *	↓*	Glycerophospholipid metabolism
Negative ion mode									
P12	Uric acid ^a^	0.61	167.0123	C_5_H_4_N_4_O_3_	1.50	↓ **	↑ **	↑ **	Purine metabolism
P13	Inosine ^a^	0.66	267.0561	C_10_H_12_N_4_O_5_	1.02	↑ **	↓ **	↓ **	Purine metabolism
P14	l-tryptophan ^a^	1.28	203.0728	C_11_H_12_N_2_O_2_	1.29	↓ **	↑ *	↑	Tryptophan metabolism
P15	Taurocholic acid ^a^	2.40	514.2839	C_26_H_45_NO_7_S	1.74	↓ **	↑	↑	Bile acid metabolism
P5	LysoPC(22:6) ^b^	6.86	612.3295	C_30_H_50_NO_7_P	1.74	↓ **	↑ **	↑	Glycerophospholipid metabolism
P6	LysoPC(18:2) ^b^	6.90	564.3299	C_26_H_50_NO_7_P	3.08	↓ **	↑ **	↑ **	Glycerophospholipid metabolism
P7	LysoPC(20:4) ^b^	6.92	588.3292	C_28_H_50_NO_7_P	2.44	↓ **	↓	↑ **	Glycerophospholipid metabolism
P8	LysoPC(16:0) ^b^	7.86	540.3302	C_24_H_50_NO_7_P	3.37	↓ *	↑ *	↑	Glycerophospholipid metabolism
P16	LysoPC(15:0) ^b^	7.87	480.3091	C_23_H_48_NO_7_P	4.33	↓ **	↑	↓	Glycerophospholipid metabolism

The M/C represents the change trend in model group compared with control group, Y/M and mKG/M represent the change trends in the positive group and mKG group compared with the model group, respectively. ^a^ Metabolites validated by reference standards; ^b^ Metabolites identified by databases. ↑: up-regulated, ↓: down-regulated; * *p* < 0.05, ** *p* < 0.01.

## Supplementary Materials

Supplementary materials can be found at www.mdpi.com/1422-0067/18/3/602/s1.

## Abbreviations

mKG	Modified Kushen Gancao
TCM	traditional Chinese medicines
OVA	ovalbumin
UPLC-Q-TOF/MS	ultra high-performance liquid chromatography coupled with quadrupole time-of-flight mass spectrometry
PLS−DA	partial least squares–discriminate analysis
OPLS−DA	orthogonal partial least squares–discriminate analysis
Ach	acetylcholine
ASM	airway smooth muscle
Dex	dexamethasone
HE	hematoxylin-eosin
BPI	base peak intensity
PCA	principal component analysis
VIP	variable importance of project
KEGG	kyoto encyclopedia of genes and genomes
HMDB	human metabolome database
DHA	docosahexaenoic acid
MAP	mitogen activated protein
DSR	dehydrosphinganine reductase
MPT	mitochondrial permeability transitionpore
AMP	adenosine monophosphate
HDM	house dust mite
AA	Arachidonic acid
PUFA	polyunsaturated fatty acid
Lo	lipoxygenase
HETE	hydroxyeicosatetraenoic acid
BALF	bronchoalveolar lavage fluid

## References

[1] Busse W.W., Lemanske R.F. (2001). Asthma. N. Engl. J. Med..

[2] Masoli M., Fabian D., Holt S., Beasley R. (2004). The global burden of asthma: Executive summary of the GINA Dissemination Committee report. Allergy.

[3] Dahlin A., Tantisira K.G. (2012). Integrative systems biology approaches in asthma pharmacogenomics. Pharmacogenomics.

[4] Drazen J.M., Silverman E.K., Lee T.H. (2000). Heterogeneity of therapeutic responses in asthma. Br. Med. Bull..

[5] Li X.M., Huang C.K., Zhang T.F., Teper A.A., Srivastava K., Schofield B.H., Sampson H.A. (2000). The Chinese herbal medicine formula MSSM-002 suppresses allergic airway hyperreactivity and modulates TH1/TH2 responses in a murine model of allergic asthma. J. Allergy Clin. Immunol..

[6] Gao Y., Yao L.F., Zhao Y., Wei L.M., Guo P., Yu M., Cao B., Li T., Chen H., Zou Z.M. (2016). The Chinese Herbal Medicine Formula mKG Suppresses Pulmonary Fibrosis of Mice Induced by Bleomycin. Int. J. Mol. Sci..

[7] He X., Fang J., Huang L., Wang J., Huang X. (2015). Sophora flavescens Ait.: Traditional usage, phytochemistry and pharmacology of an important traditional Chinese medicine. J. Ethnopharmacol..

[8] Liu C., Yang N., Song Y., Wang L., Zi J., Zhang S., Dunkin D., Busse P., Weir D., Tversky J. (2015). Ganoderic acid C1 isolated from the anti-asthma formula, ASHMI suppresses TNF-α production by mouse macrophages and peripheral blood mononuclear cells from asthma patients. Int. Immunopharmacol..

[9] Fu Q., Wang J., Ma Z., Ma S. (2014). Anti-asthmatic effects of matrine in a mouse model of allergic asthma. Fitoterapia.

[10] Zhang T.Z., Fu Q., Chen T., Ma S.P. (2015). Anti-asthmatic effects of oxymatrine in a mouse model of allergic asthma through regulating CD40 signaling. Chin. J. Nat. Med..

[11] Wang X., Zhang H., Chen L., Shan L., Fan G., Gao X. (2013). Liquorice, a unique “guide drug” of traditional Chinese medicine: A review of its role in drug interactions. J. Ethnopharmacol..

[12] Shin Y.W., Bae E.A., Lee B., Lee S.H., Kim J.A., Kim Y.S., Kim D.H. (2007). In vitro and in vivo antiallergic effects of Glycyrrhiza glabra and its components. Planta Med..

[13] Ram A., Mabalirajan U., Das M., Bhattacharya I., Dinda A.K., Gangal S.V., Ghosh B. (2006). Glycyrrhizin alleviates experimental allergic asthma in mice. Int. Immunopharmacol..

[14] Wu Q., Tang Y., Hu X., Wang Q., Lei W., Zhou L., Huang J. (2016). Regulation of Th1/Th2 balance through OX40/OX40L signalling by glycyrrhizic acid in a murine model of asthma. Respirology.

[15] Yang N., Liang B., Srivastava K., Zeng J., Zhan J., Brown L., Sampson H., Goldfarb J., Emala C., Li X.M. (2013). The Sophora flavescens flavonoid compound trifolirhizin inhibits acetylcholine induced airway smooth muscle contraction. Phytochemistry.

[16] Jin M., Zhao K., Huang Q., Xu C., Shang P. (2012). Isolation, structure and bioactivities of the polysaccharides from *Angelica sinensis* (Oliv.) Diels: A review. Carbohydr. Polym..

[17] Chao W.W., Lin B.F. (2011). Bioactivities of major constituents isolated from *Angelica sinensis* (Danggui). Chin. Med..

[18] Comhair S.A., McDunn J., Bennett C., Fettig J., Erzurum S.C., Kalhan S.C. (2015). Metabolomic Endotype of Asthma. J. Immunol. (Baltim. Md. 1950).

[19] Drexler D.M., Reily M.D., Shipkova P.A. (2011). Advances in mass spectrometry applied to pharmaceutical metabolomics. Anal. Bioanal. Chem..

[20] Liang X., Chen X., Liang Q., Zhang H., Hu P., Wang Y., Luo G. (2011). Metabonomic study of Chinese medicine Shuanglong formula as an effective treatment for myocardial infarction in rats. J. Proteome Res..

[21] Yu M., Cui F.X., Jia H.M., Zhou C., Yang Y., Zhang H.W., Ding G., Zou Z.M. (2016). Aberrant purine metabolism in allergic asthma revealed by plasma metabolomics. J. Pharm. Biomed. Anal..

[22] Stanley W.C., Recchia F.A., Lopaschuk G.D. (2005). Myocardial substrate metabolism in the normal and failing heart. Physiol. Rev..

[23] Al-Biltagi M., Isa M., Bediwy A.S., Helaly N., El Lebedy D.D. (2012). l-carnitine improves the asthma control in children with moderate persistent asthma. J. Allergy.

[24] Cui Y., Liu X., Wang M., Liu L., Sun X., Ma L., Xie W., Wang C., Tang S., Wang D. (2014). Lysophosphatidylcholine and amide as metabolites for detecting alzheimer disease using ultrahigh-performance liquid chromatography-quadrupole time-of-flight mass spectrometry-based metabonomics. J. Neuropathol. Exp. Neurol..

[25] Shang V.C., O’Sullivan S.E., Kendall D.A., Roberts R.E. (2016). The endogenous cannabinoid anandamide increases human airway epithelial cell permeability through an arachidonic acid metabolite. Pharmacol. Res..

[26] Miyata J., Arita M. (2015). Role of omega-3 fatty acids and their metabolites in asthma and allergic diseases. Allergol. Int. Off. J. Jpn. Soc. Allergol..

[27] Huang W.C., Tsai T.H., Chuang L.T., Li Y.Y., Zouboulis C.C., Tsai P.J. (2014). Anti-bacterial and anti-inflammatory properties of capric acid against Propionibacterium acnes: A comparative study with lauric acid. J. Dermatol. Sci..

[28] Rodriguez-Rodriguez E., Perea J.M., Jimenez A.I., Rodriguez-Rodriguez P., Lopez-Sobaler A.M., Ortega R.M. (2010). Fat intake and asthma in Spanish schoolchildren. Eur. J. Clin. Nutr..

[29] Hannun Y.A., Obeid L.M. (2008). Principles of bioactive lipid signalling: Lessons from sphingolipids. Nat. Rev. Mol. Cell Biol..

[30] Greenberg A.J., Hackett S.R., Harshman L.G., Clark A.G. (2011). Environmental and genetic perturbations reveal different networks of metabolic regulation. Mol. Syst. Biol..

[31] Ammit A.J., Hastie A.T., Edsall L.C., Hoffman R.K., Amrani Y., Krymskaya V.P., Kane S.A., Peters S.P., Penn R.B., Spiegel S. (2001). Sphingosine 1-phosphate modulates human airway smooth muscle cell functions that promote inflammation and airway remodeling in asthma. FASEB J. Off. Publ. Fed. Am. Soc. Exp. Biol..

[32] Shevchenko A., Simons K. (2010). Lipidomics: Coming to grips with lipid diversity. Nat. Rev. Mol. Cell Biol..

[33] Wenk M.R. (2010). Lipidomics: New tools and applications. Cell.

[34] Hatse S., De Clercq E., Balzarini J. (1999). Role of antimetabolites of purine and pyrimidine nucleotide metabolism in tumor cell differentiation. Biochem. Pharmacol..

[35] Esther C.R., Coakley R.D., Henderson A.G., Zhou Y.H., Wright F.A., Boucher R.C. (2015). Metabolomic Evaluation of Neutrophilic Airway Inflammation in Cystic Fibrosis. Chest.

[36] Van der Sluijs K.F., van de Pol M.A., Kulik W., Dijkhuis A., Smids B.S., van Eijk H.W., Karlas J.A., Molenkamp R., Wolthers K.C., Johnston S.L. (2013). Systemic tryptophan and kynurenine catabolite levels relate to severity of rhinovirus-induced asthma exacerbation: A prospective study with a parallel-group design. Thorax.

[37] Ho W.E., Xu Y.J., Cheng C., Peh H.Y., Tannenbaum S.R., Wong W.S., Ong C.N. (2014). Metabolomics Reveals Inflammatory-Linked Pulmonary Metabolic Alterations in a Murine Model of House Dust Mite-Induced Allergic Asthma. J. Proteome Res..

[38] Powell W.S., Rokach J. (2015). Biosynthesis, biological effects, and receptors of hydroxyeicosatetraenoic acids (HETEs) and oxoeicosatetraenoic acids (oxo-ETEs) derived from arachidonic acid. Biochim. Biophys. Acta.

[39] Wu X., Dev A., Leong A.B. (2003). Zileuton, a 5-lipoxygenase inhibitor, increases production of thromboxane A2 and platelet aggregation in patients with asthma. Am. J. Hematol..

[40] Jung J., Kim S.H., Lee H.S., Choi G.S., Jung Y.S., Ryu D.H., Park H.S., Hwang G.S. (2013). Serum metabolomics reveals pathways and biomarkers associated with asthma pathogenesis. Clin. Exp. Allergy J. Br. Soc. Allergy Clin. Immunol..

[41] Mattarucchi E., Baraldi E., Guillou C. (2012). Metabolomics applied to urine samples in childhood asthma; differentiation between asthma phenotypes and identification of relevant metabolites. Biomed. Chromatogr. BMC.

[42] Ho W.E., Xu Y.J., Xu F., Cheng C., Peh H.Y., Tannenbaum S.R., Wong W.S., Ong C.N. (2013). Metabolomics reveals altered metabolic pathways in experimental asthma. Am. J. Respir. Cell Mol. Biol..

[43] Liu Y.T., Jia H.M., Chang X., Ding G., Zhang H.W., Zou Z.M. (2013). The metabolic disturbances of isoproterenol induced myocardial infarction in rats based on a tissue targeted metabonomics. Mol. BioSyst..

