# Supplementary material for: The Effect of Chinese Herbal Medicine Formula mKG on Allergic Asthma by Regulating Lung and Plasma Metabolic Alternations
Source: Int J Mol Sci. 2017 Mar 10;18(3):602. doi: 10.3390/ijms18030602 (PMC5372618; doi:10.3390/ijms18030602)
Supplement: Supplementary file 1 [file ijms-18-00602-s001.pdf]

Supplementary Information

Qualitative characteristics of the chemical constituents in mKG (Sample Preparation, UPLC-Q-TOF/MS Method, and GC-MS Method)

Qualitative characteristic of chemical constituents in mKG

Sample Preparation. Aliquots of 20 mg mKG extract were transferred into a 1 mL volumetric flask which was filled to its volume capacity with initial mobile phase. The obtained solution was filtered through a 0.22 μm syringe filter before use, and 2 μL was injected into the UPLC instrument for UPLC-MS analysis. An amount of extract of the single herb equal to the same amount of the formula extract was prepared and analyzed identically to mKG. The total volatile oil of mKG was prepared in our laboratory. Briefly, 1.2 mL of Angelica oil was added to 200 g dry powder of Keushen GanCao Tang, and they were soaked together in 2000 mL of water in the steam generator for 8 h at room temperature to extract the total volatile oil. The oil was collected, dried with anhydrous sodium sulphate (yield 0.38%), and 20 μL was transferred into a tube containing 1 mL EtOAc, and then 1 μL was analyzed using GC-MS. All samples were analyzed in triplicate.

UPLC-Q-TOF/MS Method. The mKG samples were analyzed on a Waters AcquityTM Ultra Performance LC system (Waters Corporation, Milford, MA, USA) equipped with a BEH C18 column (100 mm×2.1 mm, 1.7 μm). The flow rate was 0.45 mL/min, the autosampler temperature was kept at 4 °C, and the column compartment was set at 40 °C. The mobile phase was composed of water (A) and acetonitrile (B) each containing 0.1% formic acid. The following gradient system for lung tissue samples was used: 0–0.5 min, 1% B; 0.5–5 min, 10% B; 5–13 min, 10-50% B; 13–24 min, 50–99% B; 24–27 min, washing with 99% B, and 27–30 min, equilibration with 1% B. The eluent from the column was directed to the mass spectrometer without split.

A Waters SYNAPT G2 HDMS (Waters Corp., Manchester, UK) was used to carry out the mass spectrometry with an electrospray ionization source (ESI) operating in positive ion mode. The capillary voltages were set at 3.0, sample cone voltage was 40 V, extraction cone voltage was 4.0 V, respectively. Using drying gas nitrogen, the desolvation gas rate was set to 800 L/h at 400 °C, the cone gas rate at 50 L/h, and the source temperature at 100 °C. The scan time and inter scan delay were set to 0.15 and 0.02 s, respectively. Leucine-enkephalin was used as the lockmass in all analyses (m/z 556.2771 for positive ion mode) at a concentration of 0.5 μg/mL with a flow rate of 5 μL/min. Data were collected in centroid mode from m/z 100 to m/z 1500.

GC-MS Method. The Angelica oil was analyzed by GC-MS using an Agilent computerized system 7890 gas chromatograph (Palo Alto, CA, USA), with an HP-5MS column (30 m×0.25 mm i.d.×0.25 μm film thickness) coated with 5% diphenyl and 95% dimethylpolysiloxane coupled to a 5977A mass spectrometer (Palo Alto, CA, USA). Helium carrier gas flowed at a rate of 1 mL/min, and the injector and transfer line temperatures were 250 and 300 °C, respectively. The oven temperature was maintained at 60 °C for 5 min and then increased to 140 °C at a rate of 4 °C/min. The temperature was then increased to 280 °C at a rate of 10 °C/min, and the column was maintained at this temperature for 5 min. The split ratio was 1:10, and the mass spectrometer was operated in an electron ionization mode at 70 eV. The mass range scanned from m/z 50 to m/z 500 at 2.33 s/scan for a full-scan mode.

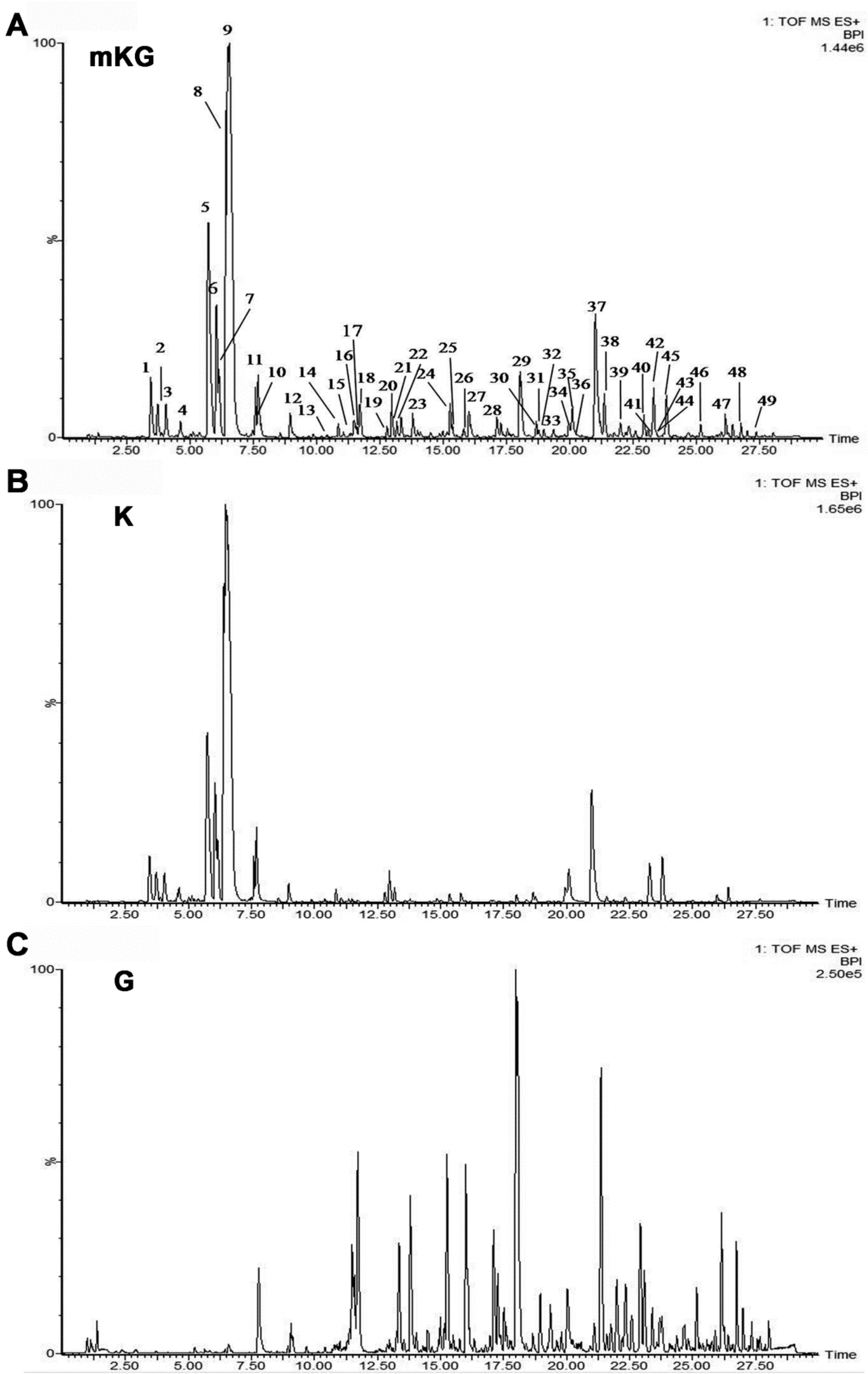

Figure S1. BPI chromatograms of mKG (**A**), Kushen (**B**), and Gancao (**C**) extract in positive and ion modes analyzed by UPLC-Q-TOF/MS.

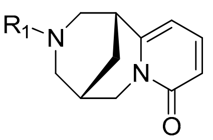

1. N-methylcytisine R_1=CH_3
2. cytisine R_1=H

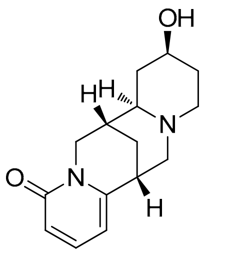

3. baptifoline

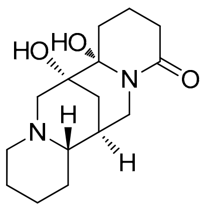

4. 6,7-dihydroxylupanine

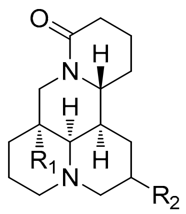

5. matrine R_1=H, R_2=H
10. 9α-Hydroxymatrine R_1=H, R_2=OH
11. 5α-Hydroxymatrine R_1=OH, R_2=H

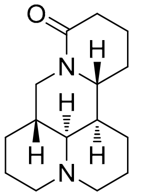

6. sophoridine

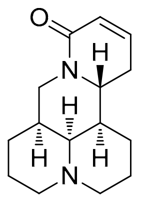

7. sophocarpine

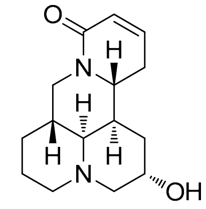

8. 9α-hydroxysophocarpine

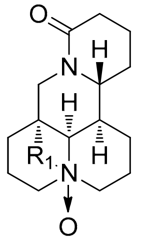

9. oxymatrine R_1=H
12. sophoranol N-oxide R_1=OH

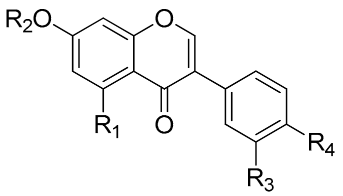

A= 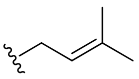

13. sophorabioside (6CI,7CI,8CI) R_1=OH, R_2=H, R_3=H, R_4=H, R_5=Man(2-6)Glu-O, R_6=H
14. lanceolarin (7CI) R_1=OH, R_2=Api(2-6)Glu, R_3=H, R_4=H, R_5=OCH_3, R_6=H
15. kakkanin R_1=OH, R_2=Xyl(2-6)Glu, R_3=H, R_4=H, R_5=OCH_3, R_6=H
20. kushenol O R_1=H, R_2=Xyl(2-6)Glu, R_3=H, R_4=H, R_5=OCH_3, R_6=H
23. formonentin R_1=H, R_2=H, R_3=H, R_4=H, R_5=OCH_3, R_6=H
25. calycosin R_1=H, R_2=H, R_3=H, R_4=H, R_5=OCH_3, R_6=OH
30. formononetin R_1=H, R_2=H, R_3=H, R_4=H, R_5=OCH_3, R_6=H
33. glicoricone R_1=H R_2=H, R_3=OCH_3, R_4=B, R_5=OCH_3, R_6=OH
46. licoricone R_1=H, R_2=H,R_3=OCH_3, R_4=B, R_5=OCH_3, R_6=OH

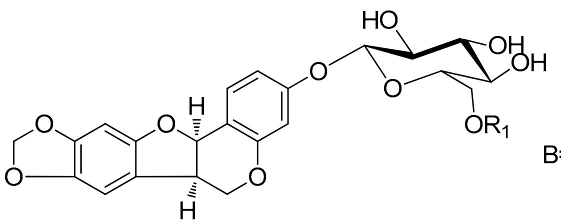

B= 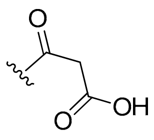

16. trifolirhizin R_1=H
26. trifolirhizin 6'-O-malonate R_1=A

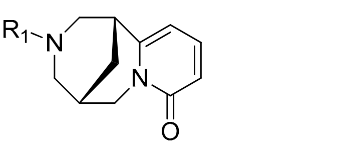

1. N-methylcytisine $R_1=CH_3$
2. cytisine $R_1=H$

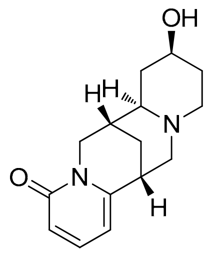

3. baptifoline

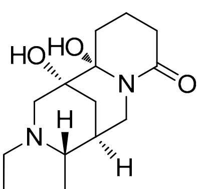

4. 6,7-dihydroxylupanine

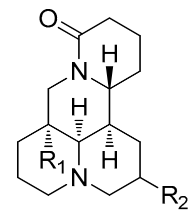

5. matrine $R_1=H$, $R_2=H$
10. 9α-Hydroxymatrine $R_1=H$, $R_2=OH$
11. 5α-Hydroxymatrine $R_1=OH$, $R_2=H$

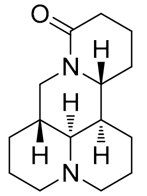

6. sophoridine

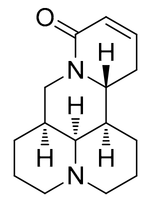

7. sophocarpine

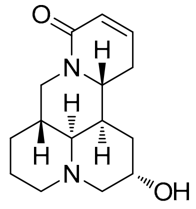

8. 9α-hydroxysophocarpine

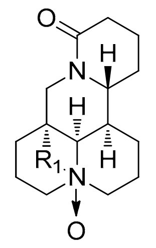

9. oxymatrine $R_1=H$
12. sophoranol N-oxide $R_1=OH$

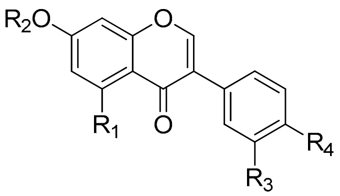

A= 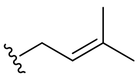

13. sophorabioside (6CI,7CI,8CI) $R_1=OH$, $R_2=H$, $R_3=H$, $R_4=H$, R_5=Man(2-6)Glu-O, $R_6=H$
14. lanceolarin (7CI) $R_1=OH$, R_2=Api(2-6)Glu, $R_3=H$, $R_4=H$, $R_5=OCH_3$, $R_6=H$
15. kakkanin $R_1=OH$, R_2=Xyl(2-6)Glu, $R_3=H$, $R_4=H$, $R_5=OCH_3$, $R_6=H$
20. kushenol O $R_1=H$, R_2=Xyl(2-6)Glu, $R_3=H$, $R_4=H$, $R_5=OCH_3$, $R_6=H$
23. formonentin $R_1=H$, $R_2=H$, $R_3=H$, $R_4=H$, $R_5=OCH_3$, $R_6=H$
25. calycosin $R_1=H$, $R_2=H$, $R_3=H$, $R_4=H$, $R_5=OCH_3$, $R_6=OH$
30. formononetin $R_1=H$, $R_2=H$, $R_3=H$, $R_4=H$, $R_5=OCH_3$, $R_6=H$
33. glicoricone $R_1=H$ $R_2=H$, $R_3=OCH_3$, $R_4=B$, $R_5=OCH_3$, $R_6=OH$
46. licoricone $R_1=H$, $R_2=H$,$R_3=OCH_3$, $R_4=B$, $R_5=OCH_3$, $R_6=OH$

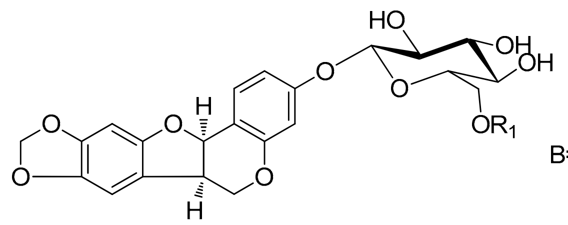

B= 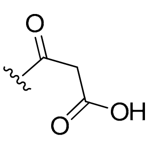

16. trifolirhizin $R_1=H$
26. trifolirhizin 6'-O-malonate $R_1=A$

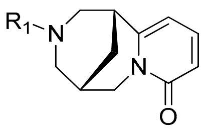

1. N-methylcytisine R_1=CH_3
2. cytisine R_1=H

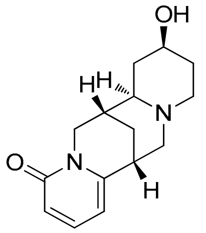

3. baptifoline

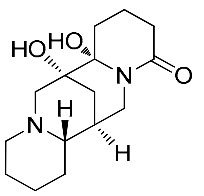

4. 6,7-dihydroxylupanine

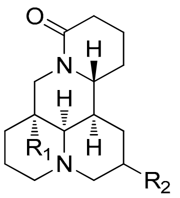

5. matrine R_1=H, R_2=H
10. 9α-Hydroxymatrine R_1=H, R_2=OH
11. 5α-Hydroxymatrine R_1=OH, R_2=H

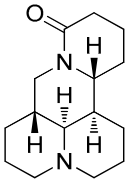

6. sophoridine

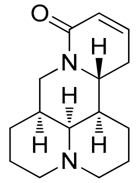

7. sophocarpine

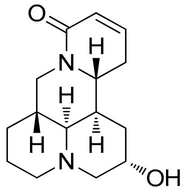

8. 9α-hydroxysophocarpine

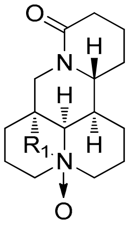

9. oxymatrine R_1=H
12. sophoranol N-oxide R_1=OH

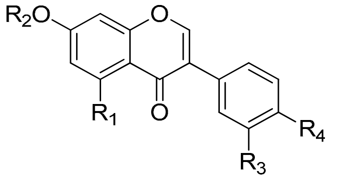

A= 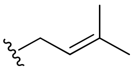

13. sophorabioside (6CI,7CI,8CI) R_1=OH, R_2=H, R_3=H, R_4=H, R_5=Man(2-6)Glu-O, R_6=H
14. lanceolarin (7CI) R_1=OH, R_2=Api(2-6)Glu, R_3=H, R_4=H, R_5=OCH_3, R_6=H
15. kakkanin R_1=OH, R_2=Xyl(2-6)Glu, R_3=H, R_4=H, R_5=OCH_3, R_6=H
20. kushenol O R_1=H, R_2=Xyl(2-6)Glu, R_3=H, R_4=H, R_5=OCH_3, R_6=H
23. formonentin R_1=H, R_2=H, R_3=H, R_4=H, R_5=OCH_3, R_6=H
25. calycosin R_1=H, R_2=H, R_3=H, R_4=H, R_5=OCH_3, R_6=OH
30. formononetin R_1=H, R_2=H, R_3=H, R_4=H, R_5=OCH_3, R_6=H
33. glicoricone R_1=H R_2=H, R_3=OCH_3, R_4=B, R_5=OCH_3, R_6=OH
46. licoricone R_1=H, R_2=H,R_3=OCH_3, R_4=B, R_5=OCH_3, R_6=OH

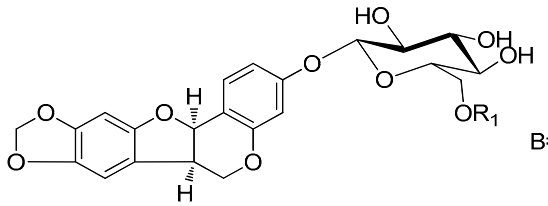

B= 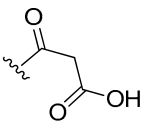

16. trifolirhizin R_1=H
26. trifolirhizin 6'-O-malonate R_1=A

Figure S2. The chemical structures of the identified compounds in the mKG preparation by UPLC-Q-TOF/MS.

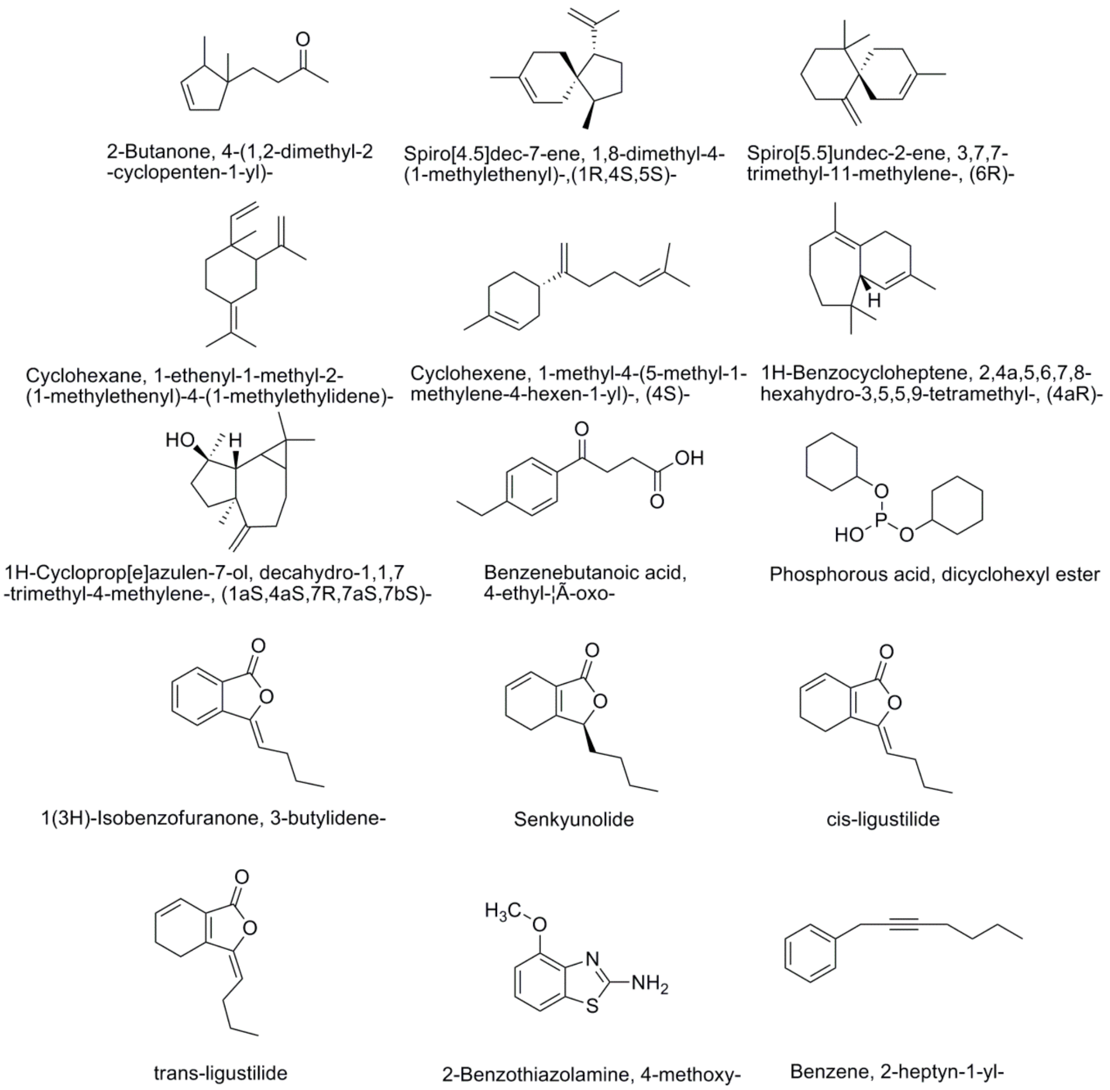

Figure S3. The chemical structures of the identified compounds in Angelica oil analyzed by using GC-MS.

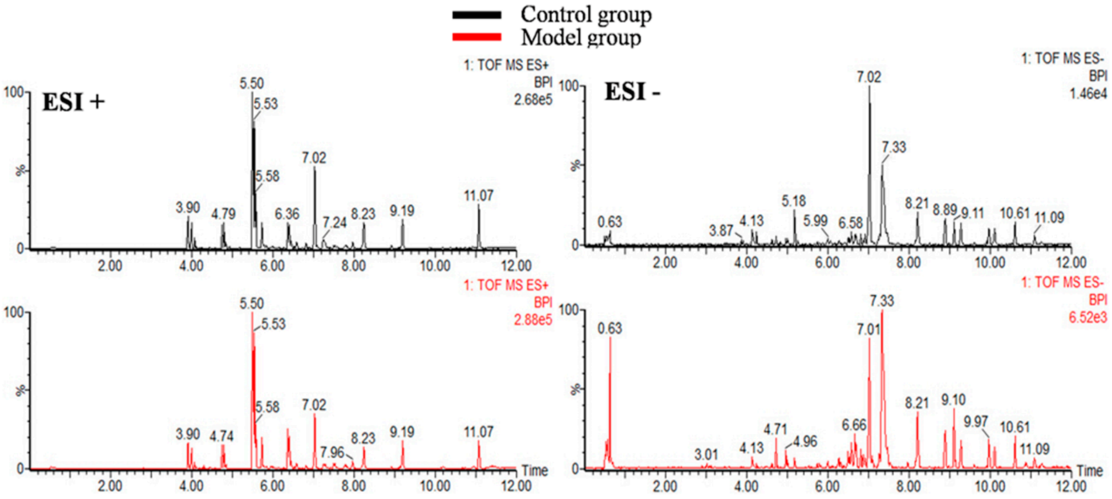

Figure S4. Typical UPLC-Q-TOF/MS base peak intensity (BPI) chromatograms of lung tissue samples from the control group and OVA-induced mice (model group) in positive ion mode and in negative ion mode.

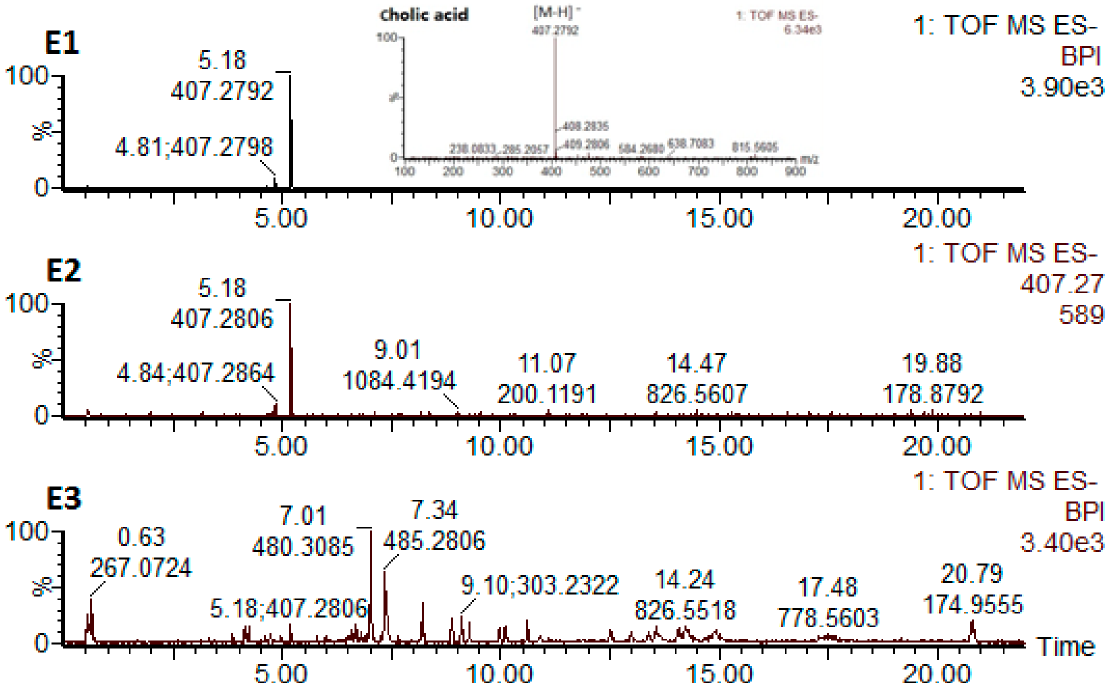

Figure S5. The UPLC-Q-TOF/MS BPI and extracted ion chromatograms of reference standard (**cholic acid**) (E1). The extracted ion chromatogram of the ion at m/z 407.2790 was shown at (E2) and typical BPI chromatograms of lung tissue samples from the QC sample in negative ion mode (E3).

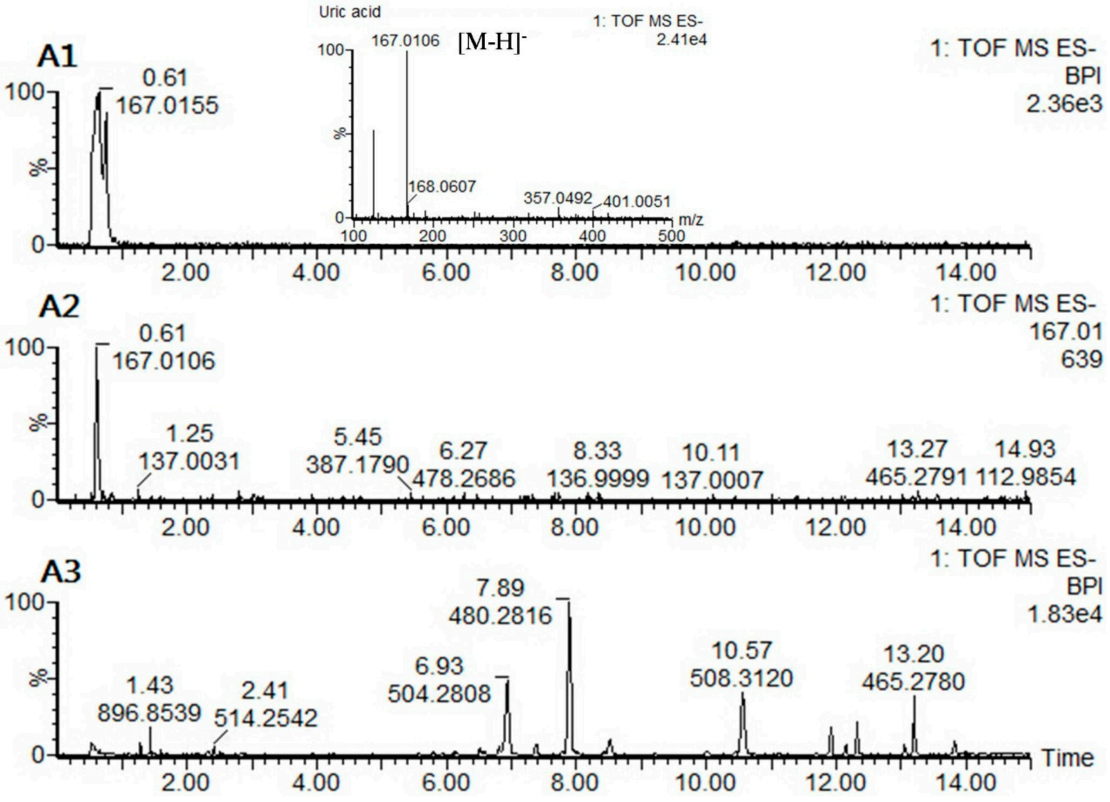

Figure S6. The UPLC-Q-TOF/MS BPI and extracted ion chromatograms of reference standard (uric acid) (A1). The extracted ion chromatogram of the ion at m/z 167.0123 was shown at (A2) and typical BPI chromatograms of plasma samples from the QC sample in negative ion mode (A3).

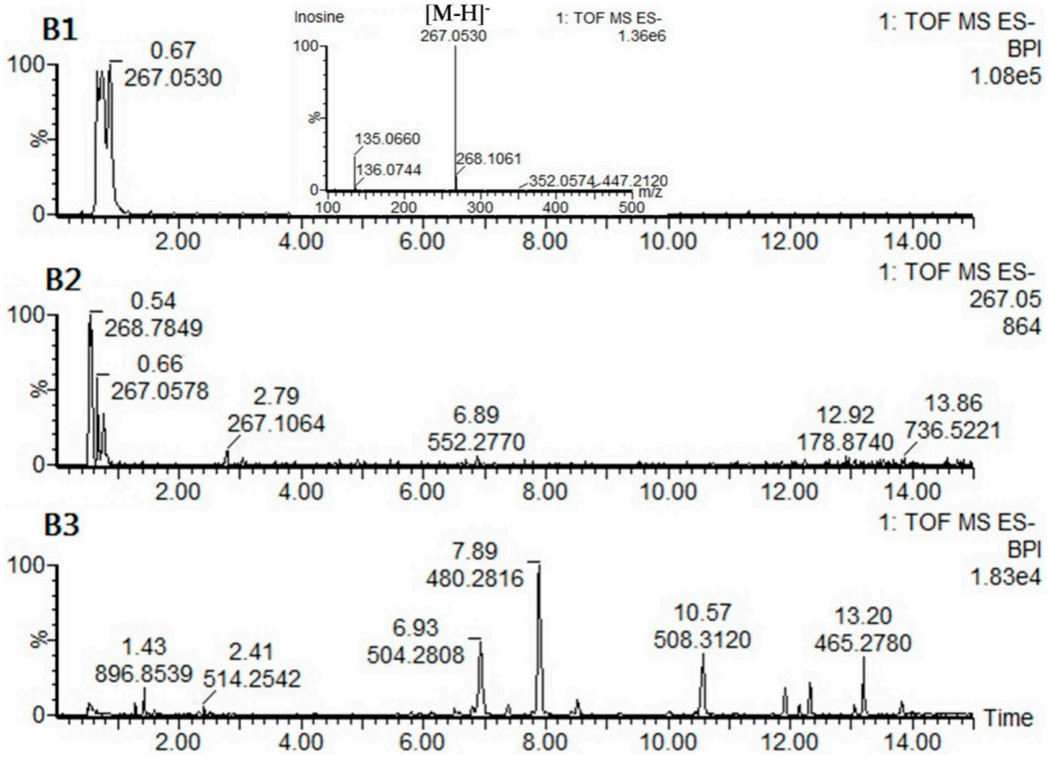

Figure S7. The UPLC-Q-TOF/MS BPI and extracted ion chromatograms of reference standard (**inosine**) (B1). The extracted ion chromatogram of the ion at m/z 267.0561 was shown at (B2) and typical BPI chromatograms of plasma samples from the QC sample in negative ion mode (B3).

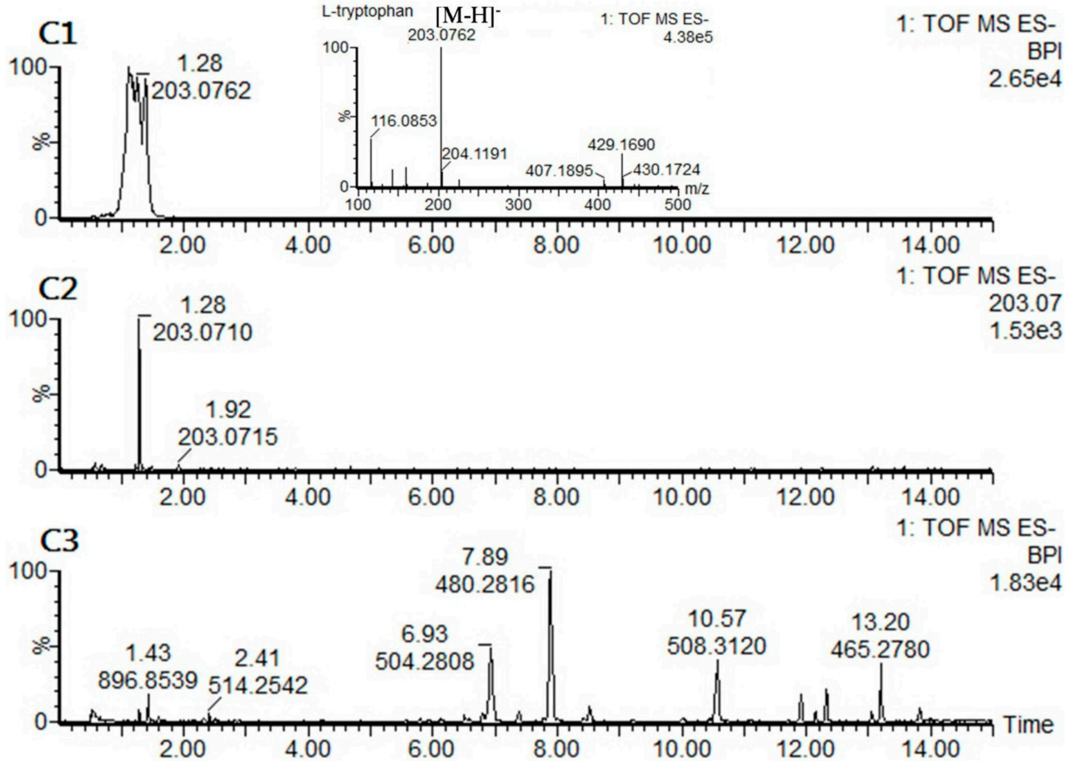

Figure S8. The UPLC-Q-TOF/MS BPI and extracted ion chromatograms of reference standard (**L-tryptophan**) (C1). The extracted ion chromatogram of the ion at m/z 203.0728 was shown at (C2) and typical BPI chromatograms of plasma samples from the QC sample in negative ion mode (C3).

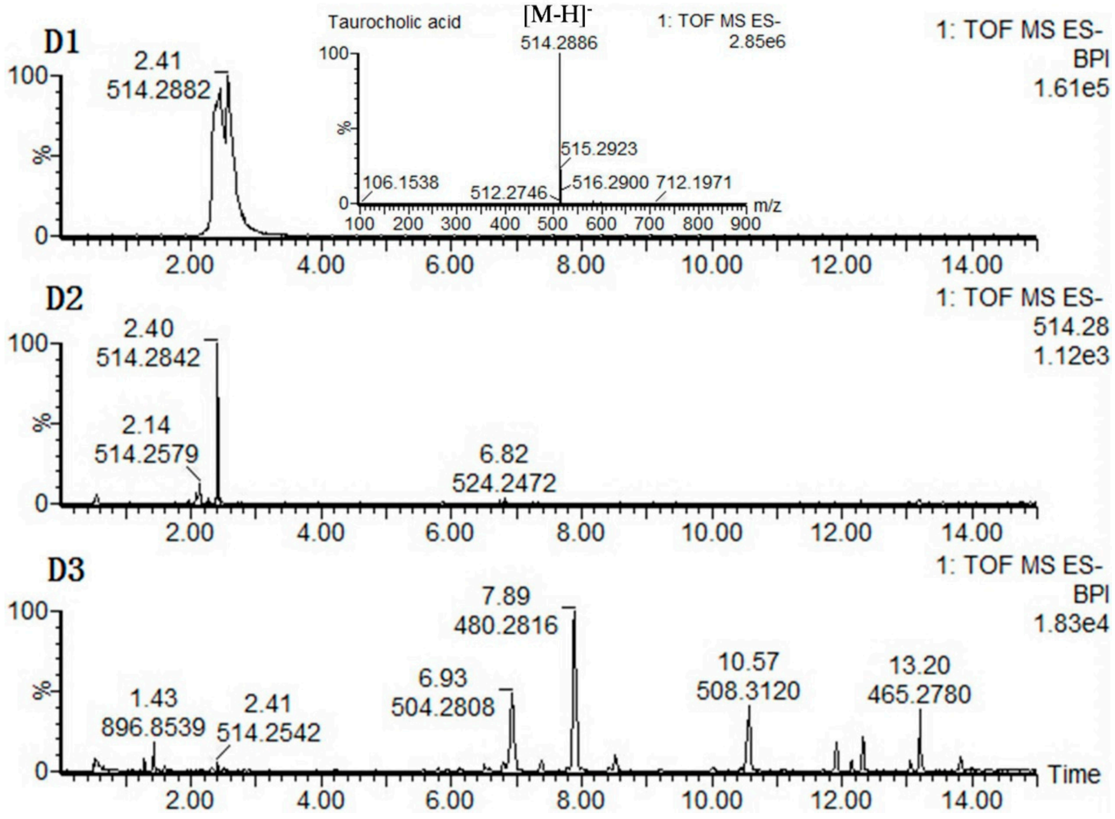

Figure S9. The UPLC-Q-TOF/MS BPI and extracted ion chromatograms of reference standard (**taurocholic acid**) (D1). The extracted ion chromatogram of ion at m/z 514.2839 was shown at (D2) and typical BPI chromatograms of plasma samples from the QC sample in negative ion mode (D3).

Table S1 The characterization of the identified compounds in mKG extract preparation by UPLC-Q-TOF/MS.

NO.	RT (min)	Identification	Formula	m/z	Error (ppm)	Biological source
1	3.45	*N*-methylcytisine	$C_{12}H_{16}N_2O$	205.1344	1.5	Sophora
2	3.71	cytosine	$C_{11}H_{14}N_2O$	191.1185	0.5	Sophora
3	4.05	baptifoline	$C_{15}H_{20}N_2O_2$	261.1609	2.3	Sophora
4	4.56	6,7-dihydroxylupanine	$C_{15}H_{20}N_2O$	281.1869	1.4	Sophora
5	5.75	matrine	$C_{15}H_{24}N_2O$	249.1967	0.0	Sophora
6	6.05	sophoridine	$C_{15}H_{24}N_2O$	249.1975	3.2	Sophora
7	6.15	sophocarpine	$C_{15}H_{22}N_2O$	247.1819	3.6	Sophora
8	6.40	9α-hydroxysophocarpine	$C_{15}H_{22}N_2O_2$	263.1770	3.8	Sophora
9	6.48	oxymatrine	$C_{15}H_{24}N_2O_2$	265.1924	3.0	Sophora
10	7.59	9α-hydroxymatrine	$C_{15}H_{24}N_2O_2$	265.1917	0.4	Sophora
11	7.70	5α-hydroxymatrine	$C_{15}H_{24}N_2O_2$	265.1921	1.9	Sophora
12	8.97	sophoranol N-oxide	$C_{15}H_{24}N_2O_3$	281.1873	2.8	Sophora
13	10.40	sophorabioside (6CI,7CI,8CI)	$C_{27}H_{30}O_{14}$	579.1710	−0.7	Sophora
14	10.85	lanceolarin (7CI)	$C_{27}H_{30}O_{14}$	579.1721	1.2	Sophora
15	11.06	kakkanin	$C_{27}H_{30}O_{14}$	579.1709	−0.9	Sophora
16	11.35	trifolirhizin	$C_{22}H_{22}O_{10}$	447.1310	4.2	Sophora
17	11.48	liquiritigen	$C_{15}H_{12}O_4$	257.0825	4.3	glycyrrhiza
18	11.72	isoliquiritigen	$C_{15}H_{12}O_4$	257.0826	4.7	glycyrrhiza
19	12.78	-	$C_{27}H_{28}O_{14}$	577.1559	0.3	Sophora
20	12.97	kushenol O	$C_{27}H_{30}O_{13}$	563.1780	2.7	Sophora
21	13.17	sophoraflavone A	$C_{27}H_{30}O_{13}$	563.1774	1.6	Sophora
22	13.35	liquiritoside	$C_{21}H_{22}O_9$	419.1335	−1.7	glycyrrhiza
23	13.79	formonentin	$C_{16}H_{12}O_4$	269.0815	0.4	glycyrrhiza
24	15.24	licoricesaponin A3	$C_{48}H_{72}O_{21}$	985.4613	−3.1	glycyrrhiza
25	15.35	calycosin	$C_{16}H_{12}O_5$	285.0765	0.7	Sophora
26	15.80	trifolirhizin 6'-O-malonate	$C_{25}H_{24}O_{13}$	555.1112	−0.5	Sophora
27	15.99	22β-acetoxyglycyrrhizin	$C_{44}H_{64}O_{18}$	881.4171	−4.5	glycyrrhiza
28	17.11	licoricesaponin G2	$C_{42}H_{62}O_{17}$	839.4065	−0.1	glycyrrhiza
29	18.00	glycyrrhizic acid	$C_{42}H_{62}O_{16}$	823.4112	−0.5	glycyrrhiza
30	18.67	formononetin	$C_{16}H_{12}O_4$	269.0812	−0.7	Sophora
31	18.76	isoxanthohumol	$C_{21}H_{22}O_5$	355.1542	−0.8	Sophora
32	18.96	glycyrrhetic acid 3-O-glucuronide	$C_{36}H_{54}O_{10}$	647.3788	−1.1	glycyrrhiza
33	19.36	glicoricone	$C_{21}H_{20}O_6$	369.1338	1.6	glycyrrhiza
34	19.94	kushenol N	$C_{26}H_{30}O_7$	455.2079	2.0	Sophora
35	20.03	1-methoxyphaseollin	$C_{21}H_{20}O_5$	353.1397	2.3	glycyrrhiza
36	20.08	kushenol I	$C_{26}H_{30}O_7$	455.2074	0.9	Sophora
37	21.00	kurarinone	$C_{26}H_{30}O_6$	439.2134	3.7	Sophora
38	21.36	glycycoumarin	$C_{21}H_{20}O_6$	369.1347	2.4	glycyrrhiza
39	22.00	glyurallin A	$C_{21}H_{20}O_5$	353.1031	-	glycyrrhiza

40	22.91	glycyrin	$C_{22}H_{22}O_6$	383.1500	1.3	glycyrrhiza
41	23.07	licochalcone A	$C_{21}H_{22}O_4$	339.1601	1.5	glycyrrhiza
42	23.29	5-O-methylkushenol C	$C_{27}H_{32}O_6$	453.2273	−0.9	Sophora
43	23.40	isolicoflavonol	$C_{20}H_{18}O_6$	355.1184	0.6	glycyrrhiza
44	23.70	glycyrol	$C_{21}H_{18}O_6$	367.1186	1.1	glycyrrhiza
45	23.79	isoglycyrol	$C_{21}H_{18}O_6$	367.1190	1.2	glycyrrhiza
46	25.16	licoricone	$C_{22}H_{22}O_6$	383.1500	1.3	glycyrrhiza
47	26.42	isokurarinone	$C_{26}H_{30}O_6$	439.2135	3.2	Sophora
48	26.74	licorisoflavan C	$C_{26}H_{30}O_5$	423.2153	−5.0	glycyrrhiza
49	26.99	1-methoxyficifolinol	$C_{26}H_{30}O_5$	423.2168	−0.7	glycyrrhiza

Table S2 The characterization of the identified compounds in Angelica oil analyzed by using GC-MS.

NO.	RT (min)	Identification	Formula	CAS#
1	12.1	2-Butanone, 4-(1,2-dimethyl-2-cyclopenten-1-yl)-	$C_{11}H_{18}O$	075698-06-5
2	12.3	Spiro[4.5]dec-7-ene, 1,8-dimethyl-4-(1-methylethenyl)-,(1R,4S,5S)-	$C_{15}H_{24}$	024048-44-0
3	12.8	Spiro[5.5]undec-2-ene, 3,7,7-trimethyl-11-methylene-, (6R)-	$C_{15}H_{24}$	018431-82-8
4	13.25	Cyclohexane, 1-ethenyl-1-methyl-2-(1-methylethenyl)-4-(1-methylethylidene)-	$C_{15}H_{24}$	003242-08-8
5	13.4	Cyclohexene, 1-methyl-4-(5-methyl-1-methylene-4-hexen-1-yl)-, (4S)-	$C_{15}H_{24}$	000495-61-4
6	14.08	1H-Benzocycloheptene, 2,4a,5,6,7,8-hexahydro-3,5,5,9-tetramethyl-, (4aR)-	$C_{15}H_{24}$	001461-03-6
7	15.2	1H-Cycloprop[e]azulen-7-ol, decahydro-1,1,7-trimethyl-4-methylene-, (1aS,4aS,7R,7aS,7bS)-	$C_{15}H_{24}O$	077171-55-2
8	16.75	Benzenebutanoic acid, 4-ethyl-γ-oxo-	$C_{12}H_{14}O_3$	049594-75-4
9	16.85	Phosphorous acid, dicyclohexyl ester	$C_{12}H_{23}O_3P$	000139-69-5
10	17.15	1(3H)-Isobenzofuranone, 3-butylidene-	$C_{12}H_{12}O_2$	000551-08-6
11	17.9	Senkyunolide	$C_{12}H_{16}O_2$	63038-10-8
12	18.24	cis-ligustilide	$C_{12}H_{14}O_2$	1000365-98-5
13	19.15	trans-ligustilide	$C_{12}H_{14}O_2$	1000365-98-8
14	21.8	2-Benzothiazolamine, 4-methoxy-	$C_8H_8N_2OS$	005464-79-9
15	23.55	Benzene, 2-heptyn-1-yl-	$C_{13}H_{16}$	054725-17-6
